# Changes in the Salivary Proteome Associated With Canine Pyometra

**DOI:** 10.3389/fvets.2020.00277

**Published:** 2020-06-11

**Authors:** Lorena Franco-Martínez, Anita Horvatić, Andrea Gelemanović, Marko Samardžija, Vladimir Mrljak, María Dolores Contreras-Aguilar, Silvia Martínez-Subiela, Roman Dąbrowski, Asta Tvarijonaviciute

**Affiliations:** ^1^Interdisciplinary Laboratory of Clinical Analysis, Interlab-UMU, Regional Campus of International Excellence ‘Campus Mare Nostrum’, University of Murcia, Murcia, Spain; ^2^Faculty of Veterinary Medicine, University of Zagreb, Zagreb, Croatia; ^3^Mediterranean Institute for Life Sciences (MedILS), Split, Croatia; ^4^Department and Clinic of Animal Reproduction, Faculty of Veterinary Medicine, University of Life Sciences in Lublin, Lublin, Poland

**Keywords:** biomarker, dog, proteomics, pyometra, saliva, tandem mass tags

## Abstract

The present study evaluated for the first time changes in the saliva proteome in bitches with pyometra through a high-throughput quantitative proteomic analysis. The aims were to explore whether saliva composition could reflect the physiopathological changes occurring in canine pyometra and to identify potential biomarkers of the disease. Saliva samples from six healthy (H) and six bitches with pyometra (P) were analyzed using tandem mass tags–based approach. Additionally, 15 samples were used for the validation of changes in haptoglobin (Hp) concentration in saliva of dogs with pyometra. Proteomic analysis quantified 707 proteins in saliva. Comparison of the two groups revealed 16 unique proteins significantly modulated in saliva, with S100A calcium-binding protein 12 (S100A12), vimentin, and Hp the most up-regulated in canine pyometra. According to PANTHER (Protein Analysis Through Evolutionary Relationships) classification tool, these proteins are mainly related to proinflammatory mediators, acute-phase proteins, and sepsis. In conclusion, it can be stated that there are changes in various proteins in saliva in canine pyometra reflecting different physiopathological changes occurring in this disease. These proteins could be a source of potential non-invasive biomarkers for this disease that should be confirmed in future studies.

## Introduction

Pyometra, which literally means “pus-filled uterus,” is the most common disease of the uterus in intact adult bitches after non-pregnant estrus cycles (1). It is a post-estrum suppurative bacterial infection of the uterus, which may cause accumulation of inflammatory exudate and produces a variety of clinical manifestations (2). Generally, the clinical signs of closed-cervix pyometra are more severe because of the presence of enlarged uterus and endotoxemia, increasing the risks of uterine rupture and the systemic inflammatory response syndrome (3). Thus, a wide range of clinical signs can be observed from subclinical to a fatal illness, which could make the early diagnosis of pyometra challenging, especially when there is no vaginal discharge (2).

Hormonal and bacterial factors are involved in the development of canine pyometra. The higher concentrations of progesterone during the luteal phase promote changes in the uterus including an increase in glandular activity and secretions, and lesser contractions and leukocyte response (4), enabling physiological pregnancy, although they are also ideal for the promotion of microbial growth and pyometra. Because estrogens increase uterine sensitivity to progesterone, the risk of pyometra is increased in older bitches or in those under estrogen therapy (5, 6). However, there is a scarcity of information regarding the complex pathogenesis of canine pyometra (2), and the knowledge of the changes that occur in saliva proteome in bitches with pyometra could help to clarify this pathogenesis and detect possible potential biomarkers of the disease.

The use of saliva provides several advantages compared to samples that are collected by invasive methods such as blood, being safer for the personnel and the patient, easy and pain-free to collect, and reducing sampling stress (7). Saliva usually presents a higher variability of proteins in comparison to serum and can be used to monitor disease status providing additional knowledge of disease pathophysiology and reveal potential new biomarkers. The salivary proteome in 36 healthy dogs was analyzed using nanoscale liquid chromatography–tandem mass spectrometry, identifying a total of 2,491 proteins (8). Because saliva may reflect the overall physiological status, it is envisioned that it will be used increasingly in relation to diseases' early diagnosis or monitoring (9). In dogs, saliva has been usefully employed for the diagnosis of infectious (10–12) and metabolic (13) diseases, among others. Saliva composition is also known to be modulated in canine pyometra because increased and decreased concentrations of adenosine deaminase and adiponectin, respectively—biomarkers related to of inflammation—were found in bitches with pyometra in comparison to healthy ones (14, 15). Therefore, a detailed overview of the possible changes in salivary protein composition could improve the understanding of the physiopathology of canine pyometra, as well as point out possible biomarkers of the disease.

Over the last years, the sensitivity of the proteomic analysis has been greatly improved by the discovery of novel technologies such as isobaric tags (16–18). Those technologies have several advantages over gel-based proteomics such as improved sensitivity and reproducibility (16–18). Tandem mass tag (TMT) allows the relative simultaneous quantification of differentially labeled peptides (19) in several biological samples. The approach has been successfully applied in the study of several canine infectious diseases such as parvovirus or leishmaniasis, among others (11, 20). Although the high time and costs of proteomics in comparison to other analysis limited their usefulness for direct routine clinical applications, these techniques are increasingly used as the initial point for the search of biomarkers. However, to the authors' best knowledge, the possible changes in salivary proteome in canine pyometra have not been yet studied.

The objective of the present study was to evaluate possible changes in the salivary proteome in bitches with pyometra compared to healthy ones. This would highly contribute to gaining knowledge about the reflection of physiopathological changes in saliva associated with the disease and the use of saliva as a source of potential non-invasive biomarkers of this disease.

## Materials and Methods

### Animals and Saliva Sampling

Twenty-seven client-owned bitches of different breeds presented to the Department and Clinic of Animal Reproduction, Faculty of Veterinary Medicine, University of Life Sciences, Lublin, Poland, from November 2018 to October 2019 were included in the study.

All animals were in diestrus, and according to health status, dogs were allocated into two groups. One group (H) consisted of bitches that were classified as healthy after a complete physical examination and hematological and serum biochemistry. The second group (P) comprised bitches diagnosed with open-cervix pyometra. This diagnosis was based on clinical examination (with all cases showing pyrexia, apathy, polydipsia-polyuria, and anorexia), hematology (with increased white blood cell count and band neutrophil counts), serum biochemistry (with increased globulins and C-reactive protein), and abdominal ultrasound findings consistent with pyometra, as described previously (14).

In the pyometra group, samples were collected at the moment of diagnosis and before any treatment; according to owners, bitches showed compatible clinical signs 0–3 days before their presentation to the Department and Clinic of Animal Reproduction. Immediately after surgery, the bacteriological examination of the pus collected from the uterus showed *Escherichia coli* in all cases.

Exclusion criteria included the presence of gingivitis or any other oral or systemic diseases, including infectious, metabolic, or endocrine. In addition, in the case of P group, bitches were not included if presented with closed-cervix pyometra.

The dogs were randomly selected in the database from those that met the inclusion criteria. Six animals of each group were selected for proteomic study (two mixed breeds, two Labrador retriever, and two German shepherd, body weight 35 ± 9.3 kg, aged 8.8 ± 2.09 years) for the H group and three mixed breeds: two German Shepherd, one Siberian husky, body weight 30.5 ± 9.7 kg, aged 9.1 ± 3.29 years for P group), whereas samples of the rest of the animals (four mixed breeds, two German Shepherd, one Bullterrier, body weight 18.0 ± 10.7 kg, aged 8.3 ± 3.45 years for H; and five mixed breeds and one of each of Dachshund, Pekinese, and Yorkshire Terrier; body weight 12.0 ± 8.9 kg, aged 8.8 ± 2.67 years for P) were used in the validation study. All the procedures were approved by the University of Lublin Institutional Animal Care and Ethics Committee (approval no. 27/2015), and written consent was obtained from the owners.

For sample collection, at least 0.5 mL of total saliva was collected from each patient by introducing a small piece of sponge in the mouth, as described elsewhere (21). When the sponges were thoroughly moistened, they were placed into collection devices (Salivette saliva collection tube/V-bottom; Sarstedt, Aktiengesellschaft & Co, Nümbrecht, Germany), centrifuged (3,000 × g for 10 min, 4°C), and the supernatant was stored at −80°C until analysis (22).

### Proteomic Study and Liquid Chromatography–Tandem Mass Spectrometry Analysis

From each sample, 35 μg of protein was subjected to reduction, alkylation, digestion, and labeling using 6-plex TMT reagents according to manufacturer instructions (Thermo Fisher Scientific, Waltham, MA USA) as described previously (23).

Dionex Ultimate 3000 RSLC nano-flow system (Dionex, Camberley, UK) and Orbitrap Q Exactive Plus mass spectrometer (Thermo Fisher Scientific) were used for the liquid chromatography–tandem mass spectrometry, as described elsewhere (23). SEQUEST algorithm, Proteome Discoverer (version 2.0., Thermo Fisher Scientific), was used for peptide identification and relative quantification. NCBI database search against *Canis lupus* FASTA files was performed considering two trypsin missed cleavage sites, precursor tolerance of 10 ppm, and fragment mass tolerance of 0.02 Da. Percolator algorithm within the Proteome Discoverer workflow was used to determine the false discovery rate (FDR) for peptide identification, which was set at 1%. In cases where GI accession number was described as unnamed, BLAST analysis and sequence comparison public database (NCBI) information were performed, and the result with the higher identity and query cover were included (>98% in all cases).

### Statistical Analysis

Proteins with fewer than two unique peptides and those with more than 90% missing data in all samples were removed from the analysis. Sample outliers were detected and removed for each group and protein using the Dixon's test from R package *outliers* v0.14 (24). Shapiro-Wilk test showed that the majority of the analyzed proteins did not follow a normal distribution; therefore, Wilcoxon-Mann-Whitney test was performed to test the difference in protein abundance between groups. Fold change between two groups was calculated as mean (P)/mean (H). Statistics were performed with R v3.2.2 (25).

Heatmap was designed using R package ggplot2 v3.1.1 (26), ggdendro v0.1-20 (27), and pheatmap v1.0.12 (28).

Genes encoding the differentially expressed proteins between H and P groups were used to determine the Gene ontology (GO) terms overrepresented in CMT using Protein Analysis Through Evolutionary Relationships (PANTHER) classification tool (http://www.pantherdb.org/).

### Validation of Haptoglobin as Salivary Biomarker for Canine Pyometra

Haptoglobin (Hp) in saliva was selected for validation because it showed 2-fold higher concentrations in bitches with pyometra in comparison to healthy ones in the proteomic analysis.

Haptoglobin was measured by a time-resolved fluorometry-based immunoassay described elsewhere and validated for its use for canine saliva (29). Haptoglobin concentrations were expressed in μg/mL.

## Results

### High-Resolution Quantitative Proteomic Analysis

**Figure 1:**
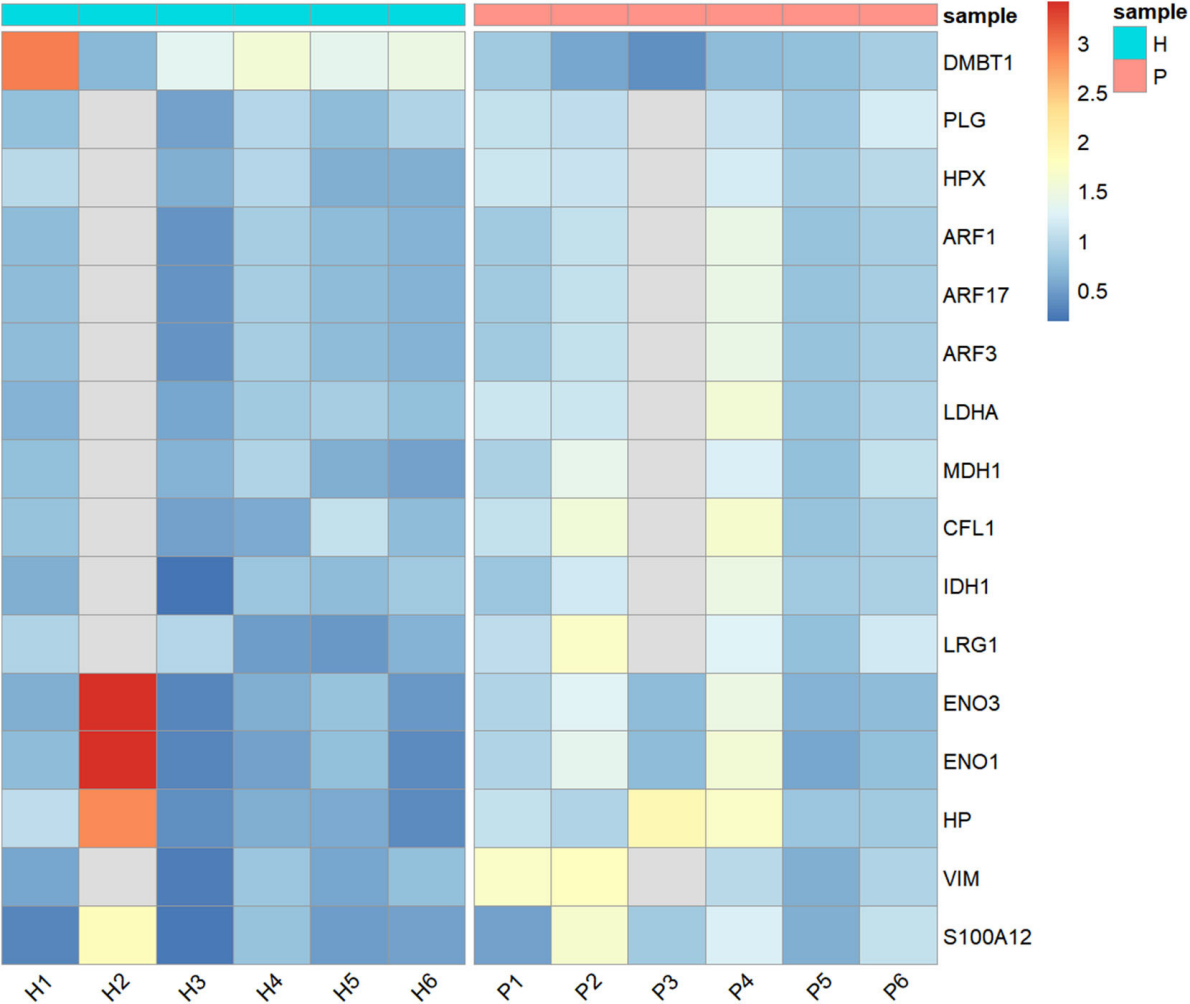
Heatmap showing the relative abundance (color) of salivary proteins in healthy (H) and bitches with pyometra (P).

After the removal of proteins with fewer than two unique peptides, missing data, outliers, and <5% FDR, 707 proteins remained to be analyzed (Supplementary Table 1). Wilcoxon-Mann-Whitney test pointed out statistically significantly different abundance between H and P groups in 16 unique proteins, as shown in Table 1 and Figure 1. Of those, only one protein named DMBT-1 (deleted in malignant brain tumors 1) was down-regulated, whereas 15 were up-regulated in canine pyometra.

**Table 1:** In saliva, there were 16 proteins identified with significantly different abundance between bitches with pyometra and healthy controls.

**GI accession**	**Gene symbol**	**Description**	***P***	**Fold change**	**Median (25th−75th interquartile) healthy**	**Median (25th−75th interquartile) pyometra**
**Proteins downregulated in pyometra when compared to HC**						
928186547	DMBT1	Deleted in malignant brain tumors 1 protein–like	0.026	0.437	1.403 (1.289–1.518)	0.724 (0.608–0.841)
**Proteins upregulated in pyometra when compared to HC**						
558695388	PLG	Plasminogen precursor	0.032	1.343	0.731 (0.624–0.838)	1.061 (1.026–1.096)
73988725	HPX	Hemopexin	0.032	1.403	0.615 (0.431–0.8)	1.085 (1.02–1.15)
1005261202	ARF1	ADP–ribosylation factor 1	0.040	1.484	0.708 (0.676–0.74)	0.878 (0.753–1.003)
1418330752	ARF17	ADP-ribosylation factor–like 17–like	0.040	1.484	0.708 (0.676–0.74)	0.878 (0.753–1.003)
545545650	ARF3	ADP-ribosylation factor 3	0.040	1.484	0.708 (0.676–0.74)	0.878 (0.753–1.003)
545536994	LDHA	L-lactate dehydrogenase A chain	0.032	1.510	0.732 (0.65–0.814)	1.117 (1.023–1.211)
57092971	MDH1	Malate dehydrogenase, cytoplasmic	0.032	1.538	0.644 (0.588–0.7)	1.078 (0.912–1.245)
1418315363	CFL1	Cofilin-1–like	0.040	1.624	0.722 (0.632–0.813)	1.051 (0.719–1.383)
89573987	IDH1	Isocitrate dehydrogenase 1	0.032	1.647	0.707 (0.601–0.813)	0.889 (0.723–1.055)
1418218016	LRG1	Leucine-rich alpha-2-glycoprotein	0.032	1.698	0.647 (0.423–0.872)	1.145 (1.025–1.265)
928134045	ENO3	Beta-enolase	0.030	1.729	0.601 (0.522–0.681)	0.832 (0.589–1.076)
345800677	ENO1	Alpha-enolase	0.030	1.859	0.524 (0.349–0.7)	0.839 (0.577–1.102)
258499	HP	Haptoglobin heavy chain	0.030	2.047	0.573 (0.452–0.695)	0.993 (0.65–1.337)
559098393	VIM	Vimentin	0.032	2.069	0.559 (0.465–0.654)	0.991 (0.612–1.371)
1418313614	S100A12	Protein S100-A12–like	0.017	2.149	0.478 (0.392–0.565)	0.942 (0.691–1.194)

These differentially expressed proteins in saliva between H and P groups were used for qualitative analysis in terms of functional clusters, according to the PANTHER classification system (http://www.pantherdb.org). The identified differentially modulated proteins between H and P had two molecular functions: catalytic activity (80%) and binding (20%). Six different biological processes were involved, being metabolic and cellular process the majoritarian with 39.9 and 33.3% of proteins, respectively. Six biological pathways were involved, being blood coagulation, integrin signaling pathway, and plasminogen activating cascade the most represented with 22.2% of proteins each. Finally, in relation with protein class, 41% were hydrolases, followed by oxidoreductases (25%).

### Validation of Haptoglobin in Saliva

When Hp was measured in saliva, it was significantly higher in bitches with canine pyometra [median (25th−75th percentile) [3.09 (1.39–10.24) μg/mL] in comparison to healthy ones [0.24 (0.19–0.61) μg/mL] (*p* < 0.001).

## Discussion

This is the first study in which salivary proteome was evaluated in bitches with pyometra and compared to healthy controls, showing 16 proteins differentially expressed between the two groups that reflect the activation of different biological pathways and that can potentially be of use as non-invasive biomarkers of this disease. To the best of the authors' knowledge, most of these proteins are described for the first time in saliva in canine pyometra.

S100A12, vimentin, and Hp were the proteins most up-regulated in canine pyometra. S100A proteins interact with endothelium cells, mononuclear phagocytes, and lymphocytes, participating in the immune cell activation and the generation of proinflammatory mediator (30). In line with our findings, a marked upregulation of S100A12 gene expression in the uterus of bitches with canine pyometra in comparison to healthy controls has been reported (31). Also, in humans, S100A12 has been proposed as a feasible biomarker of diagnostic and prognostic in sepsis, showing higher plasma concentration in patients with deadly septic shock in comparison to healthy individuals and survivor patients (32), and showed high sensitivity and specificity as serum biomarker of neonatal sepsis (33). Vimentin is a type of intermediate filament protein that is up-regulated during epithelial-to-mesenchymal transition, a process that occurs during neural development, wound healing, and cancer metastasis (34). Recently, it has been associated with sepsis (35), and therefore, this could explain high concentrations of vimentin found in saliva in a pyometra group.

Haptoglobin is considered a moderate acute-phase protein in dogs, increasing its concentrations ~2- to 5-fold in serum under inflammation (36). Increases in serum Hp have been reported in a variety of diseases of the dog including heartworm (37) or leishmaniasis (38). Serum Hp has been also proven of use for monitoring the postoperative period in bitches with pyometra (39). The results of the proteomic analysis in relation with salivary Hp concentrations were further verified by a time-resolved fluorometry-based immunoassay for its use in canine saliva, showing an increase in Hp in saliva of bitches with pyometra in comparison to healthy ones. Our results are in line with a previous report in which Hp in saliva increases in a group of dogs with different diseases including three cases of canine pyometra (29).

Finally, the differentially modulated proteins between H and P were mainly related to catalytic activity (80%) and binding (20%) molecular functions. Catalytic activity was represented by proteins including Hp, plasminogen, and hemopexin. According to PANTHER classification systems, these proteins participate in important roles such as regulation of cell death, inflammatory response, blood coagulation, and metabolic processes, respectively. On the other hand, binding molecular function was represented by ADP-ribosylation factors, which are G proteins that participate in the intracellular and vesicle-mediated transport. The knowledge of the biological roles of the proteins in saliva affected by canine pyometra could be of utility for the increased knowledge of the pathophysiology of the disease and could point out novel insights for diagnostic, prognostic, or monitoring biomarkers, as well as potential therapeutic targets.

The relatively small sample size used is one of the main limitations of this report, and therefore, this study should be considered as a pilot study. However, the sample size is higher than the minimum recommended of three replicates (40) and is in concordance with similar proteomic studies (41). Second, some of the proteins differently modulated in saliva in canine pyometra are not specific to this condition, and therefore, their possible value as confirmatory biomarkers for diagnosis of pyometra would be questionable. However, their use as prognostic indicators or for monitoring response to treatment might be worth further research in the future. Additionally, because only bitches without oral alterations including gingivitis were included, the applications of these biomarkers in clinical situations where oral alterations are present should be performed with caution. Finally, in order to use a more homogeneous group, only open cervix pyometra cases were included in the study. However, in the future, it would be of interest to evaluate also close pyometra and explore if in these cases there could be specific markers that could contribute to their detection.

In conclusion, it can be stated that there are changes in various proteins in saliva in canine pyometra reflecting different physiopathological changes occurring in this disease. These proteins could be a source of potential non-invasive biomarkers for this disease that should be confirmed in future studies.

## Data Availability Statement

The mass spectrometry proteomics data have been deposited to the ProteomeXchange Consortium via the PRIDE partner repository with the dataset identifier PXD018692.

## Ethics Statement

The animal study was reviewed and approved by University of Lublin Institutional Animal Care and Ethics Committee (approval number 27/2015). Written informed consent was obtained from the owners for the participation of their animals in this study.

## Author Contributions

LF-M analyzed the specimens, analyzed the data, and wrote the manuscript. AH, MC-A, and MS performed the proteomic study. AG performed the statistical analyses and created tables and figures. AT, SM-S and VM conceived and designed research. RD collected the samples. All authors read, correct, and approved the manuscript.

## Conflict of Interest

The authors declare that the research was conducted in the absence of any commercial or financial relationships that could be construed as a potential conflict of interest.
