# Corrigendum: Changes in the Salivary Proteome Associated With Canine Pyometra

**DOI:** 10.3389/fvets.2020.629913

**Published:** 2020-12-14

**Authors:** Lorena Franco-Martínez, Anita Horvatić, Andrea Gelemanović, Marko Samardžija, Vladimir Mrljak, María Dolores Contreras-Aguilar, Silvia Martínez-Subiela, Roman Dabrowski, Asta Tvarijonaviciute

**Affiliations:** ^1^Interdisciplinary Laboratory of Clinical Analysis, Interlab-UMU, Regional Campus of International Excellence ‘Campus Mare Nostrum’, University of Murcia, Murcia, Spain; ^2^Faculty of Veterinary Medicine, University of Zagreb, Zagreb, Croatia; ^3^Mediterranean Institute for Life Sciences (MedILS), Split, Croatia; ^4^Department and Clinic of Animal Reproduction, Faculty of Veterinary Medicine, University of Life Sciences in Lublin, Lublin, Poland

**Keywords:** biomarker, dog, proteomics, pyometra, saliva, tandem mass tags

In the published article, there was an error in Affiliation **1**. Instead of “Interdisciplinary Laboratory of Clinical Pathology, Interlab-UMU, University of Murcia, Murcia, Spain,” it should be:

“Interdisciplinary Laboratory of Clinical Analysis, Interlab-UMU, Regional Campus of International Excellence ‘Campus Mare Nostrum', University of Murcia, Murcia, Spain”.

The authors apologize for this error and state that this does not change the scientific conclusions of the article in any way. The original article has been updated.

