# Supplementary material for: Changes in the Salivary Proteome Associated With Canine Pyometra
Source: Front Vet Sci. 2020 Jun 11;7:277. doi: 10.3389/fvets.2020.00277 (PMC7300179; doi:10.3389/fvets.2020.00277)
Supplement: Supplementary file 1 [file Table_1.DOC]

**Supplementary Table 1. Salivary proteins identified in dogs with pyometra and healthy controls.**

| **Protein Name** | **Accession Number** | **No. Unique Peptides** | **Protein function** | **Fold-change (log2)** | **P** |
| --- | --- | --- | --- | --- | --- |
| S100A12 | 1418313614 | 8 | protein S100-A12-like [Canis lupus dingo] | 1,104 | 0,017 |
| DMBT1 | 928186547 | 3 | deleted in malignant brain tumors 1 protein-like, partial [Canis lupus familiaris] | -1,196 | 0,026 |
| ENO1 | 345800677 | 14 | LOW QUALITY PROTEIN: alpha-enolase [Canis lupus familiaris] | 0,894 | 0,030 |
| ENO3 | 928134045 | 4 | beta-enolase isoform X1 [Canis lupus familiaris] | 0,790 | 0,030 |
| ENO3 | 1418313159 | 4 | beta-enolase isoform X2 [Canis lupus dingo] | 0,790 | 0,030 |
| HP | 258499 | 16 | haptoglobin heavy chain, HpH chain [dogs, Peptide, 245 aa] | 1,034 | 0,030 |
| HP | 73957095 | 19 | haptoglobin-like [Canis lupus familiaris] | 1,026 | 0,030 |
| HP | 123511 | 19 | RecName: Full=Haptoglobin; Contains: RecName: Full=Haptoglobin alpha chain; Contains: RecName: Full=Haptoglobin beta chain | 1,026 | 0,030 |
| HPX | 73988725 | 9 | hemopexin [Canis lupus familiaris] | 0,488 | 0,032 |
| IDH1 | 89573987 | 3 | isocitrate dehydrogenase 1, partial [Canis lupus familiaris] | 0,720 | 0,032 |
| LDHA | 1418223224 | 9 | L-lactate dehydrogenase A chain isoform X1 [Canis lupus dingo] | 0,595 | 0,032 |
| LDHA | 545536994 | 9 | L-lactate dehydrogenase A chain isoform X2 [Canis lupus familiaris] | 0,595 | 0,032 |
| LRG1 | 1418218016 | 9 | leucine-rich alpha-2-glycoprotein [Canis lupus dingo] | 0,764 | 0,032 |
| MDH1 | 57092971 | 9 | malate dehydrogenase, cytoplasmic [Canis lupus familiaris] | 0,621 | 0,032 |
| PLG | 558695388 | 7 | plasminogen precursor [Canis lupus familiaris] | 0,426 | 0,032 |
| VIM | 559098393 | 3 | vimentin [Canis lupus familiaris] | 1,049 | 0,032 |
| ARF1 | 1418213778 | 3 | ADP-ribosylation factor 1 [Canis lupus dingo] | 0,569 | 0,040 |
| ARF1 | 1005261202 | 3 | ADP-ribosylation factor 1 [Canis lupus familiaris] | 0,569 | 0,040 |
| ARF17 | 1418330752 | 3 | ADP-ribosylation factor-like 17-like [Canis lupus dingo] | 0,569 | 0,040 |
| ARF3 | 545545650 | 3 | ADP-ribosylation factor 3 [Canis lupus familiaris] | 0,569 | 0,040 |
| CFL2 | 1418315363 | 6 | cofilin-1-like [Canis lupus dingo] | 0,700 | 0,040 |
| DMBT1 | 928175938 | 15 | deleted in malignant brain tumors 1 protein isoform X1 [Canis lupus familiaris] | -0,858 | 0,041 |
| DMBT1 | 928175949 | 15 | deleted in malignant brain tumors 1 protein isoform X2 [Canis lupus familiaris] | -0,858 | 0,041 |
| DMBT1 | 928175953 | 15 | deleted in malignant brain tumors 1 protein isoform X3 [Canis lupus familiaris] | -0,858 | 0,041 |
| DMBT1 | 1418246878 | 14 | LOW QUALITY PROTEIN: deleted in malignant brain tumors 1 protein [Canis lupus dingo] | -0,849 | 0,041 |
| ENO1 | 1418310052 | 17 | alpha-enolase isoform X1 [Canis lupus dingo] | 0,881 | 0,052 |
| ENO1 | 1418310054 | 15 | alpha-enolase isoform X2 [Canis lupus dingo] | 0,866 | 0,052 |
| N/A | 16607675 | 7 | unnamed protein product [Canis lupus familiaris] | 0,749 | 0,056 |
| CAP1 | 1418293878 | 11 | adenylyl cyclase-associated protein 1 isoform X1 [Canis lupus dingo] | 0,281 | 0,056 |
| CAP1 | 1418293884 | 10 | adenylyl cyclase-associated protein 1 isoform X2 [Canis lupus dingo] | 0,278 | 0,056 |
| FGB | 1418336465 | 10 | fibrinogen beta chain [Canis lupus dingo] | 0,560 | 0,056 |
| GSTO1 | 1418245560 | 8 | glutathione S-transferase omega-1 [Canis lupus dingo] | 0,718 | 0,056 |
| LDHA | 1418345245 | 5 | L-lactate dehydrogenase A chain-like [Canis lupus dingo] | 0,586 | 0,056 |
| LDHA | 545507351 | 5 | L-lactate dehydrogenase A chain-like [Canis lupus familiaris] | 0,586 | 0,056 |
| LDHA | 1239955652 | 3 | L-lactate dehydrogenase A chain-like isoform X1 [Canis lupus familiaris] | 0,760 | 0,056 |
| LDHA | 1239958986 | 4 | LOW QUALITY PROTEIN: L-lactate dehydrogenase A chain-like [Canis lupus familiaris] | 0,538 | 0,056 |
| MMP9 | 50978992 | 8 | matrix metalloproteinase-9 precursor [Canis lupus familiaris] | 0,486 | 0,056 |
| PRDX6 | 1418316704 | 9 | peroxiredoxin-6 [Canis lupus dingo] | 0,597 | 0,056 |
| PRDX6 | 1239911065 | 9 | peroxiredoxin-6 [Canis lupus familiaris] | 0,597 | 0,056 |
| UBE2N | 1418299948 | 2 | ubiquitin-conjugating enzyme E2 N [Canis lupus dingo] | 0,547 | 0,056 |
| UBE2N | 1239937430 | 2 | ubiquitin-conjugating enzyme E2 N isoform X1 [Canis lupus familiaris] | 0,547 | 0,056 |
| UBE2N | 1239937432 | 2 | ubiquitin-conjugating enzyme E2 N isoform X2 [Canis lupus familiaris] | 0,547 | 0,056 |
| UBE2N | 1418327462 | 2 | ubiquitin-conjugating enzyme E2 N-like [Canis lupus dingo] | 0,547 | 0,056 |
| UBE2N | 1418510313 | 2 | ubiquitin-conjugating enzyme E2 N-like [Canis lupus dingo] | 0,547 | 0,056 |
| UBE2N | 345799601 | 2 | ubiquitin-conjugating enzyme E2 N-like [Canis lupus familiaris] | 0,547 | 0,056 |
| MUC4 | 1418200086 | 13 | mucin-4 [Canis lupus dingo] | 0,298 | 0,063 |
| MUC4 | 1239976879 | 13 | mucin-4 [Canis lupus familiaris] | 0,298 | 0,063 |
| SERPINB1 | 1239979297 | 8 | leukocyte elastase inhibitor [Canis lupus familiaris] | 0,655 | 0,063 |
| ENO2 | 1418256156 | 2 | gamma-enolase [Canis lupus dingo] | 0,464 | 0,082 |
| HBA1 | 1101972892 | 6 | TPA: globin A1 [Canis lupus familiaris] | 0,949 | 0,082 |
| HBB | 227343817 | 9 | Chain B, Crystal Structure Of Dog (Canis Familiaris) Hemoglobin | 1,065 | 0,082 |
| HBB | 1418222274 | 8 | hemoglobin subunit beta [Canis lupus dingo] | 1,065 | 0,082 |
| LOC476315 | 928125111 | 13 | LOW QUALITY PROTEIN: alpha-enolase-like [Canis lupus familiaris] | 0,898 | 0,082 |
| S100A9 | 928140605 | 9 | protein S100-A9 [Canis lupus familiaris] | 0,824 | 0,082 |
| KLK1 | 1418303522 | 3 | kallikrein-1-like isoform X1 [Canis lupus dingo] | -0,760 | 0,093 |
| KLK1 | 1418303524 | 3 | kallikrein-1-like isoform X2 [Canis lupus dingo] | -0,760 | 0,093 |
| N/A | 16607718 | 8 | unnamed protein product [Canis lupus familiaris] | 0,763 | 0,095 |
| ADH4 | 74002001 | 2 | alcohol dehydrogenase 4 [Canis lupus familiaris] | 0,555 | 0,095 |
| ALB | 6687188 | 2 | albumin [Canis lupus familiaris] | -0,796 | 0,095 |
| ALB | 1104685307 | 2 | Chain A, Serum Albumin | -0,796 | 0,095 |
| BASP1 | 1239899708 | 2 | brain acid soluble protein 1 [Canis lupus familiaris] | 0,490 | 0,095 |
| C4A | 1239928570 | 11 | complement C4-A [Canis lupus familiaris] | 0,907 | 0,095 |
| C4A | 1418290456 | 11 | complement C4-A-like [Canis lupus dingo] | 0,907 | 0,095 |
| CORO1A | 345801939 | 4 | coronin-1A [Canis lupus familiaris] | 1,117 | 0,095 |
| COTL1 | 1418309542 | 5 | coactosin-like protein [Canis lupus dingo] | 0,478 | 0,095 |
| COTL1 | 359319594 | 5 | coactosin-like protein, partial [Canis lupus familiaris] | 0,478 | 0,095 |
| DSG3 | 1418314248 | 4 | desmoglein-3 [Canis lupus dingo] | 0,382 | 0,095 |
| FETUB | 74003556 | 5 | fetuin-B [Canis lupus familiaris] | 0,622 | 0,095 |
| FGG | 545524897 | 9 | fibrinogen gamma chain isoform X1 [Canis lupus familiaris] | 0,382 | 0,095 |
| FGG | 73977992 | 9 | fibrinogen gamma chain isoform X2 [Canis lupus familiaris] | 0,382 | 0,095 |
| GDA | 1418301814 | 4 | guanine deaminase [Canis lupus dingo] | 0,892 | 0,095 |
| HBA | 194368499 | 5 | Chain C, Hemoglobin Subunit Alpha | 0,728 | 0,095 |
| HBA | 399567840 | 5 | hemoglobin subunit alpha-like [Canis lupus familiaris] | 0,728 | 0,095 |
| HBA | 399567842 | 5 | hemoglobin subunit alpha-like [Canis lupus familiaris] | 0,728 | 0,095 |
| IDH1 | 74005287 | 4 | isocitrate dehydrogenase [NADP] cytoplasmic [Canis lupus familiaris] | 0,630 | 0,095 |
| IDH1 | 1418342146 | 4 | LOW QUALITY PROTEIN: isocitrate dehydrogenase [NADP] cytoplasmic [Canis lupus dingo] | 0,630 | 0,095 |
| IGH | 208342048 | 3 | immunoglobulin heavy chain variable region, partial [Canis lupus familiaris] | 0,551 | 0,095 |
| IGH-10 | 1494245227 | 8 | immunoglobulin heavy chain IGH-10 [Canis lupus familiaris] | 0,727 | 0,095 |
| IGH-12 | 1494245231 | 8 | immunoglobulin heavy chain IGH-12 [Canis lupus familiaris] | 0,727 | 0,095 |
| IGH-15 | 1494245237 | 8 | immunoglobulin heavy chain IGH-15 [Canis lupus familiaris] | 0,727 | 0,095 |
| IGH-16 | 1494245239 | 8 | immunoglobulin heavy chain IGH-16 [Canis lupus familiaris] | 0,727 | 0,095 |
| IGH-17 | 1494245241 | 8 | immunoglobulin heavy chain IGH-17 [Canis lupus familiaris] | 0,727 | 0,095 |
| IGH-3 | 1494245213 | 7 | immunoglobulin heavy chain IGH-3 [Canis lupus familiaris] | 0,738 | 0,095 |
| IGH-5 | 1494245217 | 8 | immunoglobulin heavy chain IGH-5 [Canis lupus familiaris] | 0,661 | 0,095 |
| IGH-7 | 1494245221 | 8 | immunoglobulin heavy chain IGH-7 [Canis lupus familiaris] | 0,727 | 0,095 |
| IGHAC | 46392569 | 2 | immunoglobulin alpha heavy chain constant regin variant C, partial [Canis lupus familiaris] | 1,447 | 0,095 |
| PFN1 | 1239901610 | 4 | profilin-1 [Canis lupus familiaris] | 0,651 | 0,095 |
| SERPINC1 | 359320010 | 4 | antithrombin-III [Canis lupus familiaris] | 0,555 | 0,095 |
| YWHAB | 1418252030 | 5 | 14-3-3 protein beta/alpha [Canis lupus dingo] | 0,529 | 0,095 |
| YWHAS | 1418505824 | 12 | 14-3-3 protein sigma [Canis lupus dingo] | 0,543 | 0,095 |
| N/A | 1418322788 | 4 | alpha-1-acid glycoprotein 1-like [Canis lupus dingo] | 0,469 | 0,111 |
| N/A | 1239979503 | 5 | LOW QUALITY PROTEIN: uncharacterized protein LOC106558262 [Canis lupus familiaris] | 0,748 | 0,111 |
| N/A | 1418253519 | 5 | LOW QUALITY PROTEIN: uncharacterized protein LOC112674420 [Canis lupus dingo] | 0,748 | 0,111 |
| MMP9 | 2564101 | 10 | gelatinase B [Canis lupus familiaris] | 0,312 | 0,111 |
| MMP9 | 1418252189 | 10 | matrix metalloproteinase-9 [Canis lupus dingo] | 0,312 | 0,111 |
| MMP9 | 11034716 | 10 | matrix metalloproteinase-9 [Canis lupus familiaris] | 0,312 | 0,111 |
| CANF1 | 1418264299 | 14 | major allergen Can f 1 [Canis lupus dingo] | 0,528 | 0,126 |
| HBB | 1418222276 | 5 | hemoglobin subunit beta-like [Canis lupus dingo] | 0,959 | 0,126 |
| HBD | 103484123 | 2 | globin, partial [Canis lupus familiaris] | 0,974 | 0,126 |
| LCN1; OBP2B | 3121745 | 14 | RecName: Full=Major allergen Can f 1; AltName: Full=Allergen Dog 1; AltName: Allergen=Can f 1; Flags: Precursor | 0,528 | 0,126 |
| S100A8 | 224969390 | 6 | S100 calcium binding protein A8 [Canis lupus familiaris] | 0,877 | 0,126 |
| N/A | 124847 | 4 | RecName: Full=Double-headed protease inhibitor, submandibular gland | 1,131 | 0,132 |
| CRISP2 | 545519428 | 8 | cysteine-rich secretory protein 2 isoform X1 [Canis lupus familiaris] | -0,873 | 0,132 |
| CRISP2 | 1239929902 | 8 | cysteine-rich secretory protein 2 isoform X2 [Canis lupus familiaris] | -0,873 | 0,132 |
| KLK1 | 1418303494 | 9 | kallikrein-1 isoform X1 [Canis lupus dingo] | -0,600 | 0,132 |
| KLK1 | 1418303496 | 9 | kallikrein-1 isoform X2 [Canis lupus dingo] | -0,600 | 0,132 |
| KLK1 | 55741639 | 8 | kallikrein-1 precursor [Canis lupus familiaris] | -0,602 | 0,132 |
| A1BG | 1418297237 | 7 | alpha-1B-glycoprotein [Canis lupus dingo] | 0,306 | 0,151 |
| A1BG | 545487024 | 7 | alpha-1B-glycoprotein [Canis lupus familiaris] | 0,306 | 0,151 |
| ALDOA | 1418280689 | 11 | fructose-bisphosphate aldolase A [Canis lupus dingo] | 0,747 | 0,151 |
| C3 | 1418218430 | 46 | complement C3 [Canis lupus dingo] | 0,358 | 0,151 |
| C3 | 1239951704 | 42 | complement C3 [Canis lupus familiaris] | 0,364 | 0,151 |
| EEF1A1 | 928133169 | 2 | elongation factor 1-alpha 1-like [Canis lupus familiaris] | 1,061 | 0,151 |
| EEF2 | 1418240337 | 10 | elongation factor 2 [Canis lupus dingo] | 0,502 | 0,151 |
| FETUB | 1418515534 | 3 | fetuin-B [Canis lupus dingo] | 0,637 | 0,151 |
| FN1 | 1418343247 | 5 | fibronectin isoform X1 [Canis lupus dingo] | 0,473 | 0,151 |
| FN1 | 928182521 | 5 | fibronectin isoform X1 [Canis lupus familiaris] | 0,473 | 0,151 |
| FN1 | 1418343265 | 5 | fibronectin isoform X10 [Canis lupus dingo] | 0,473 | 0,151 |
| FN1 | 928182523 | 5 | fibronectin isoform X10 [Canis lupus familiaris] | 0,473 | 0,151 |
| FN1 | 1418343267 | 5 | fibronectin isoform X11 [Canis lupus dingo] | 0,473 | 0,151 |
| FN1 | 928182519 | 5 | fibronectin isoform X11 [Canis lupus familiaris] | 0,473 | 0,151 |
| FN1 | 1418343249 | 5 | fibronectin isoform X2 [Canis lupus dingo] | 0,473 | 0,151 |
| FN1 | 1239982239 | 5 | fibronectin isoform X2 [Canis lupus familiaris] | 0,473 | 0,151 |
| FN1 | 1418343251 | 5 | fibronectin isoform X3 [Canis lupus dingo] | 0,473 | 0,151 |
| FN1 | 1239982241 | 5 | fibronectin isoform X3 [Canis lupus familiaris] | 0,473 | 0,151 |
| FN1 | 1418343253 | 5 | fibronectin isoform X4 [Canis lupus dingo] | 0,473 | 0,151 |
| FN1 | 928182507 | 5 | fibronectin isoform X4 [Canis lupus familiaris] | 0,473 | 0,151 |
| FN1 | 1418343255 | 5 | fibronectin isoform X5 [Canis lupus dingo] | 0,473 | 0,151 |
| FN1 | 928182509 | 5 | fibronectin isoform X5 [Canis lupus familiaris] | 0,473 | 0,151 |
| FN1 | 1418343257 | 5 | fibronectin isoform X6 [Canis lupus dingo] | 0,473 | 0,151 |
| FN1 | 1239982243 | 5 | fibronectin isoform X6 [Canis lupus familiaris] | 0,473 | 0,151 |
| FN1 | 1418343259 | 5 | fibronectin isoform X7 [Canis lupus dingo] | 0,473 | 0,151 |
| FN1 | 928182511 | 5 | fibronectin isoform X7 [Canis lupus familiaris] | 0,473 | 0,151 |
| FN1 | 1418343261 | 5 | fibronectin isoform X8 [Canis lupus dingo] | 0,473 | 0,151 |
| FN1 | 1239982245 | 5 | fibronectin isoform X8 [Canis lupus familiaris] | 0,473 | 0,151 |
| FN1 | 1418343263 | 5 | fibronectin isoform X9 [Canis lupus dingo] | 0,473 | 0,151 |
| FN1 | 928182513 | 5 | fibronectin isoform X9 [Canis lupus familiaris] | 0,473 | 0,151 |
| HSP70 | 17298186 | 7 | heat shock protein 70 [Canis lupus familiaris] | 0,501 | 0,151 |
| HSP70 | 56749085 | 7 | RecName: Full=Heat shock 70 kDa protein 1 | 0,501 | 0,151 |
| HSP90B1 | 159794950 | 3 | Chain G, Endoplasmin | 0,507 | 0,151 |
| IGH | 1340236572 | 2 | immunoglobulin heavy chain variable region, partial [Canis lupus familiaris] | 0,667 | 0,151 |
| LOC100856160 | 928181068 | 5 | uncharacterized protein LOC100856160 [Canis lupus familiaris] | 0,731 | 0,151 |
| LOC112674397 | 1418253487 | 5 | uncharacterized protein LOC112674397 isoform X2 [Canis lupus dingo] | 0,731 | 0,151 |
| LOC112674463 | 1418253569 | 5 | uncharacterized protein LOC112674463 [Canis lupus dingo] | 0,731 | 0,151 |
| LOC488306 | 928180958 | 5 | LOW QUALITY PROTEIN: uncharacterized protein LOC488306 [Canis lupus familiaris] | 0,731 | 0,151 |
| MMP8 | 345799783 | 3 | neutrophil collagenase [Canis lupus familiaris] | 0,475 | 0,151 |
| MMP9 | 4689268 | 2 | gelatinase B, partial [Canis lupus familiaris] | 0,534 | 0,151 |
| MMP9 | 4868451 | 4 | type IV collagenase MMP-9, partial [Canis lupus familiaris] | 0,419 | 0,151 |
| MYH9 | 1418195836 | 6 | myosin-9 [Canis lupus dingo] | 0,879 | 0,151 |
| MYH9 | 122135145 | 6 | RecName: Full=Myosin-9; AltName: Full=Myosin heavy chain 9; AltName: Full=Myosin heavy chain, non-muscle IIa; AltName: Full=Non-muscle myosin heavy chain IIa; Short=NMMHC II-a; Short=NMMHC-IIA | 0,879 | 0,151 |
| PLS3 | 1239983604 | 16 | plastin-3 [Canis lupus familiaris] | 0,452 | 0,151 |
| PLS3 | 1418318928 | 16 | plastin-3 isoform X1 [Canis lupus dingo] | 0,452 | 0,151 |
| PPIA | 1418202786 | 2 | peptidyl-prolyl cis-trans isomerase A isoform X2 [Canis lupus dingo] | 0,745 | 0,151 |
| TALDO1 | 359321944 | 13 | transaldolase [Canis lupus familiaris] | 0,615 | 0,151 |
| TKT | 545533393 | 27 | transketolase [Canis lupus familiaris] | 0,359 | 0,151 |
| TKT | 1418216286 | 27 | transketolase isoform X1 [Canis lupus dingo] | 0,359 | 0,151 |
| TKT | 1418216288 | 27 | transketolase isoform X2 [Canis lupus dingo] | 0,359 | 0,151 |
| UPP1 | 1418511026 | 7 | uridine phosphorylase 1 [Canis lupus dingo] | 0,571 | 0,151 |
| UPP1 | 1239883228 | 7 | uridine phosphorylase 1-like [Canis lupus familiaris] | 0,571 | 0,151 |
| YWHAZ | 928151832 | 10 | 14-3-3 protein zeta/delta [Canis lupus familiaris] | 0,526 | 0,151 |
| YWHAZ | 1418511015 | 9 | 14-3-3 protein zeta/delta-like [Canis lupus dingo] | 0,539 | 0,151 |
| GLUL | 158430851 | 5 | Chain A, Glutamine Synthetase | 0,821 | 0,167 |
| GLUL | 648216199 | 5 | glutamine synthetase isoform 1 [Canis lupus familiaris] | 0,821 | 0,167 |
| GLUL | 648216006 | 5 | glutamine synthetase isoform 2 [Canis lupus familiaris] | 0,821 | 0,167 |
| PSAP | 545495176 | 12 | prosaposin isoform X1 [Canis lupus familiaris] | -0,302 | 0,167 |
| PSAP | 545495178 | 12 | prosaposin isoform X2 [Canis lupus familiaris] | -0,302 | 0,167 |
| PSAP | 73952852 | 12 | prosaposin isoform X3 [Canis lupus familiaris] | -0,302 | 0,167 |
| PSAP | 545495181 | 12 | prosaposin isoform X4 [Canis lupus familiaris] | -0,302 | 0,167 |
| PSAP | 545495183 | 12 | prosaposin isoform X5 [Canis lupus familiaris] | -0,302 | 0,167 |
| PSAP | 1418325065 | 12 | prosaposin isoform X6 [Canis lupus dingo] | -0,302 | 0,167 |
| AMY2A | 74010186 | 15 | pancreatic alpha-amylase [Canis lupus familiaris] | 0,400 | 0,175 |
| N/A | 1418320657 | 15 | allergen Fel d 4-like [Canis lupus dingo] | 0,380 | 0,177 |
| N/A | 1374502923 | 15 | Chain D, Lipocalin-Can f 6 allergen | 0,380 | 0,177 |
| CANF2 | 296863542 | 10 | Chain A, Crystal Structure Of The Dog Lipocalin Allergen Can F 2 And Implications For Cross-Reactivity To The Cat Allergen Fel D 4 | 0,642 | 0,177 |
| CANF2 | 1418266307 | 10 | minor allergen Can f 2 [Canis lupus dingo] | 0,642 | 0,177 |
| CANF2 | 29292274 | 8 | precursor Can f II, partial [Canis lupus familiaris] | 0,629 | 0,177 |
| CANF2 | 29292272 | 10 | precursor Can f II, partial [Canis lupus familiaris] | 0,642 | 0,177 |
| CANF2 | 3121746 | 10 | RecName: Full=Minor allergen Can f 2; AltName: Full=Allergen Dog 2; AltName: Allergen=Can f 2; Flags: Precursor | 0,642 | 0,177 |
| KLK1 | 264597 | 2 | tissue kallikrein A beta-chain, CPK-A beta-chain {N-terminal} {EC 3.4.21.35} [dogs, pancreas, Peptide Partial, 38 aa] | -0,511 | 0,180 |
| KLK1 | 264599 | 2 | tissue kallikrein B beta-chain, CPK-B beta-chain {N-terminal} {EC 3.4.21.35} [dogs, pancreas, Peptide Partial, 39 aa] | -0,511 | 0,180 |
| CNDP2 | 1418298743 | 3 | cytosolic non-specific dipeptidase [Canis lupus dingo] | 0,261 | 0,190 |
| GAPDH | 89573935 | 2 | glyceraldehyde-3-phosphate dehydrogenase, partial [Canis lupus familiaris] | 0,375 | 0,190 |
| KRT1 | 1418221463 | 6 | keratin, type II cytoskeletal 1 [Canis lupus dingo] | 0,343 | 0,190 |
| KRT1 | 75062693 | 6 | RecName: Full=Keratin, type II cytoskeletal 1; AltName: Full=Cytokeratin-1; Short=CK-1; AltName: Full=Epithelial keratin-1; AltName: Full=Keratin-1; Short=K1; AltName: Full=Type-II keratin Kb1 | 0,343 | 0,190 |
| KRT13 | 1077167933 | 12 | keratin, type I cytoskeletal 13 [Canis lupus familiaris] | 0,533 | 0,190 |
| OLFM4 | 1239956251 | 9 | olfactomedin-4 [Canis lupus familiaris] | 0,329 | 0,190 |
| SERPINB1 | 1418205376 | 13 | leukocyte elastase inhibitor [Canis lupus dingo] | 0,345 | 0,190 |
| N/A | 1418213575 | 2 | antimicrobial peptide NK-lysin-like [Canis lupus dingo] | 1,794 | 0,200 |
| N/A | 73980904 | 2 | antimicrobial peptide NK-lysin-like [Canis lupus familiaris] | 1,794 | 0,200 |
| N/A | 74009438 | 2 | antimicrobial peptide NK-lysin-like [Canis lupus familiaris] | 1,794 | 0,200 |
| N/A | 1239941037 | 2 | antimicrobial peptide NK-lysin-like [Canis lupus familiaris] | 1,794 | 0,200 |
| ACTN2 | 1418509128 | 2 | alpha-actinin-2 isoform X1 [Canis lupus dingo] | 0,821 | 0,200 |
| ACTN2 | 1418509130 | 2 | alpha-actinin-2 isoform X2 [Canis lupus dingo] | 0,821 | 0,200 |
| B2M | 548923914 | 2 | beta-2-microglobulin precursor [Canis lupus familiaris] | -0,665 | 0,200 |
| CD109 | 1418348456 | 4 | CD109 antigen isoform X1 [Canis lupus dingo] | 0,394 | 0,200 |
| CD109 | 1239930298 | 4 | CD109 antigen isoform X1 [Canis lupus familiaris] | 0,394 | 0,200 |
| CD109 | 1418348458 | 3 | CD109 antigen isoform X2 [Canis lupus dingo] | 0,463 | 0,200 |
| CD109 | 1239930300 | 3 | CD109 antigen isoform X2 [Canis lupus familiaris] | 0,463 | 0,200 |
| CFB | 1418290466 | 3 | complement factor B [Canis lupus dingo] | 1,026 | 0,200 |
| CFB | 345778397 | 3 | complement factor B [Canis lupus familiaris] | 1,026 | 0,200 |
| CTSB | 345790427 | 3 | cathepsin B isoform X1 [Canis lupus familiaris] | 0,467 | 0,200 |
| CTSB | 1239962827 | 2 | cathepsin B isoform X2 [Canis lupus familiaris] | 0,254 | 0,200 |
| EEF1A1 | 1418199787 | 4 | LOW QUALITY PROTEIN: elongation factor 1-alpha 1-like [Canis lupus dingo] | 1,786 | 0,200 |
| EEF1A2 | 1418253120 | 3 | elongation factor 1-alpha 2 [Canis lupus dingo] | 1,786 | 0,200 |
| EEF1A2 | 1239959012 | 2 | elongation factor 1-alpha 2 [Canis lupus familiaris] | 1,786 | 0,200 |
| EEF1AL3 | 1418240311 | 2 | putative elongation factor 1-alpha-like 3 [Canis lupus dingo] | 1,786 | 0,200 |
| FAM3B | 345795447 | 2 | protein FAM3B [Canis lupus familiaris] | 0,705 | 0,200 |
| FAM3B | 1418228533 | 2 | protein FAM3B, partial [Canis lupus dingo] | 0,705 | 0,200 |
| FAM3D | 1418216695 | 3 | protein FAM3D isoform X1 [Canis lupus dingo] | 0,149 | 0,200 |
| FAM3D | 1239949597 | 3 | protein FAM3D isoform X1 [Canis lupus familiaris] | 0,149 | 0,200 |
| FAM3D | 1418216697 | 3 | protein FAM3D isoform X2 [Canis lupus dingo] | 0,149 | 0,200 |
| FAM3D | 359322299 | 3 | protein FAM3D isoform X2 [Canis lupus familiaris] | 0,149 | 0,200 |
| FAM3D | 1418216699 | 3 | protein FAM3D isoform X3 [Canis lupus dingo] | 0,149 | 0,200 |
| FAM3D | 545533257 | 3 | protein FAM3D isoform X3 [Canis lupus familiaris] | 0,149 | 0,200 |
| GSTA2 | 1418292147 | 4 | glutathione S-transferase A2 [Canis lupus dingo] | 1,313 | 0,200 |
| GSTA3 | 635545472 | 4 | glutathione S-transferase alpha 3 [Canis lupus familiaris] | 1,313 | 0,200 |
| GSTA3 | 649656024 | 4 | glutathione s-transferase alpha 3 [Canis lupus familiaris] | 1,313 | 0,200 |
| GSTM3 | 1239908410 | 5 | glutathione S-transferase Mu 3 isoform X1 [Canis lupus familiaris] | 1,001 | 0,200 |
| GSTM3 | 57088159 | 6 | glutathione S-transferase Mu 3 isoform X2 [Canis lupus familiaris] | 1,001 | 0,200 |
| LOC611632 | 1418510295 | 23 | ceruloplasmin-like [Canis lupus dingo] | 1,724 | 0,200 |
| LOC611632 | 345788999 | 22 | ceruloplasmin-like [Canis lupus familiaris] | 1,774 | 0,200 |
| NME1 | 545510194 | 3 | nucleoside diphosphate kinase A isoform X1 [Canis lupus familiaris] | 1,244 | 0,200 |
| NPEPPS | 1418337649 | 4 | puromycin-sensitive aminopeptidase isoform X1 [Canis lupus dingo] | 1,148 | 0,200 |
| NPEPPS | 545511022 | 4 | puromycin-sensitive aminopeptidase isoform X1 [Canis lupus familiaris] | 1,148 | 0,200 |
| NPEPPS | 1418337651 | 4 | puromycin-sensitive aminopeptidase isoform X2 [Canis lupus dingo] | 1,148 | 0,200 |
| NPEPPS | 1418337655 | 4 | puromycin-sensitive aminopeptidase isoform X4 [Canis lupus dingo] | 1,148 | 0,200 |
| PLG | 18139619 | 4 | plasminogen, partial [Canis lupus familiaris] | 0,827 | 0,200 |
| PREP | 1418500854 | 2 | prolyl endopeptidase [Canis lupus dingo] | 2,087 | 0,200 |
| PREP | 1239930837 | 2 | prolyl endopeptidase [Canis lupus familiaris] | 2,087 | 0,200 |
| PSMA7 | 42529469 | 2 | proteasome alpha subunit type 7, partial [Canis lupus familiaris] | 1,044 | 0,200 |
| PSMA7 | 1418252934 | 2 | proteasome subunit alpha type-7 [Canis lupus dingo] | 1,044 | 0,200 |
| PSMA7 | 928169909 | 2 | proteasome subunit alpha type-7 [Canis lupus familiaris] | 1,044 | 0,200 |
| RAD23B | 1418320433 | 2 | UV excision repair protein RAD23 homolog B [Canis lupus dingo] | 0,527 | 0,200 |
| SERPINB10 | 1418298925 | 3 | serpin B10 [Canis lupus dingo] | 1,434 | 0,200 |
| SERPINB10 | 73945839 | 3 | serpin B10 [Canis lupus familiaris] | 1,434 | 0,200 |
| THBS1 | 1418259722 | 3 | thrombospondin-1 [Canis lupus dingo] | -0,316 | 0,200 |
| THBS1 | 345794639 | 3 | thrombospondin-1 [Canis lupus familiaris] | -0,316 | 0,200 |
| VCL | 305657831 | 4 | metavinculin variant, partial [Canis lupus familiaris] | 1,219 | 0,200 |
| VCL | 345798988 | 5 | vinculin [Canis lupus familiaris] | 1,533 | 0,200 |
| VCL | 1418326635 | 5 | vinculin isoform X1 [Canis lupus dingo] | 1,533 | 0,200 |
| VCL | 1418326637 | 5 | vinculin isoform X2 [Canis lupus dingo] | 1,533 | 0,200 |
| VCP | 1418320363 | 3 | transitional endoplasmic reticulum ATPase [Canis lupus dingo] | 0,887 | 0,200 |
| VCP | 1239927670 | 3 | transitional endoplasmic reticulum ATPase isoform X2 [Canis lupus familiaris] | 0,887 | 0,200 |
| YWHAQ | 1418242530 | 2 | 14-3-3 protein theta-like [Canis lupus dingo] | 0,583 | 0,200 |
| UBE2L3 | 73995921 | 2 | ubiquitin-conjugating enzyme E2 L3 isoform X1 [Canis lupus familiaris] | 0,540 | 0,206 |
| UBE2L3 | 545544912 | 2 | ubiquitin-conjugating enzyme E2 L3 isoform X2 [Canis lupus familiaris] | 0,540 | 0,206 |
| N/A | 1239931268 | 6 | proteoglycan 4-like [Canis lupus familiaris] | 0,282 | 0,222 |
| ACTN4 | 1418305407 | 25 | alpha-actinin-4 isoform X1 [Canis lupus dingo] | 0,444 | 0,222 |
| ACTN4 | 1418305409 | 24 | alpha-actinin-4 isoform X2 [Canis lupus dingo] | 0,401 | 0,222 |
| ACTN4 | 1418305411 | 26 | alpha-actinin-4 isoform X3 [Canis lupus dingo] | 0,445 | 0,222 |
| ACTN4 | 73947736 | 23 | alpha-actinin-4 isoform X4 [Canis lupus familiaris] | 0,439 | 0,222 |
| ACTN4 | 1418305415 | 22 | alpha-actinin-4 isoform X5 [Canis lupus dingo] | 0,390 | 0,222 |
| AMY2A | 74012369 | 11 | pancreatic alpha-amylase-like [Canis lupus familiaris] | 0,516 | 0,222 |
| ARHGDIA | 73964747 | 7 | rho GDP-dissociation inhibitor 1 [Canis lupus familiaris] | 0,293 | 0,222 |
| ARHGDIB | 57106959 | 5 | rho GDP-dissociation inhibitor 2 [Canis lupus familiaris] | 0,699 | 0,222 |
| CAPG | 1418211040 | 8 | macrophage-capping protein isoform X1 [Canis lupus dingo] | 0,491 | 0,222 |
| CAPG | 545528000 | 8 | macrophage-capping protein isoform X1 [Canis lupus familiaris] | 0,491 | 0,222 |
| CAPG | 1418211046 | 6 | macrophage-capping protein isoform X2 [Canis lupus dingo] | 0,517 | 0,222 |
| CAPG | 928158112 | 6 | macrophage-capping protein isoform X2 [Canis lupus familiaris] | 0,517 | 0,222 |
| CAPNS1 | 1418298162 | 3 | calpain small subunit 1 isoform X1 [Canis lupus dingo] | 0,524 | 0,222 |
| CAPNS1 | 73947828 | 3 | calpain small subunit 1 isoform X1 [Canis lupus familiaris] | 0,524 | 0,222 |
| CAPNS1 | 1418298164 | 3 | calpain small subunit 1 isoform X2 [Canis lupus dingo] | 0,524 | 0,222 |
| CAPNS1 | 1418298168 | 3 | calpain small subunit 1 isoform X3 [Canis lupus dingo] | 0,524 | 0,222 |
| CFL1 | 57099669 | 9 | cofilin-1 [Canis lupus familiaris] | 0,490 | 0,222 |
| CSTB | 1418228302 | 2 | cystatin-B [Canis lupus dingo] | 0,393 | 0,222 |
| CTSD | 1418206780 | 3 | cathepsin D [Canis lupus dingo] | -0,052 | 0,222 |
| CTSD | 71043798 | 3 | cathepsin D precursor [Canis lupus familiaris] | -0,052 | 0,222 |
| DSC2 | 3413469 | 13 | desmocollin type 2, partial [Canis lupus familiaris] | 0,240 | 0,222 |
| DSC2 | 1418314263 | 13 | desmocollin-2 isoform X2 [Canis lupus dingo] | 0,240 | 0,222 |
| DSC2 | 545506022 | 13 | desmocollin-2 isoform X2 [Canis lupus familiaris] | 0,240 | 0,222 |
| DSC2 | 1418314267 | 13 | desmocollin-2 isoform X4 [Canis lupus dingo] | 0,240 | 0,222 |
| DSC2 | 545506026 | 13 | desmocollin-2 isoform X4 [Canis lupus familiaris] | 0,240 | 0,222 |
| EEF1A1 | 308199425 | 6 | elongation factor 1-alpha 1 [Canis lupus familiaris] | 1,017 | 0,222 |
| EEF1A1 | 1239928312 | 6 | elongation factor 1-alpha 1 isoform X1 [Canis lupus familiaris] | 1,017 | 0,222 |
| EEF1A1 | 28415560 | 4 | eukaryotic elongation factor 1-alpha, partial [Canis lupus familiaris] | 0,934 | 0,222 |
| ELANE | 1418220639 | 5 | neutrophil elastase [Canis lupus dingo] | 0,610 | 0,222 |
| ELANE | 50979246 | 5 | neutrophil elastase precursor [Canis lupus familiaris] | 0,610 | 0,222 |
| EZR | 558757359 | 11 | ezrin [Canis lupus familiaris] | 0,502 | 0,222 |
| EZR | 1418300273 | 7 | ezrin isoform X2 [Canis lupus dingo] | 0,526 | 0,222 |
| FGA | 1418336346 | 6 | fibrinogen alpha chain [Canis lupus dingo] | 0,400 | 0,222 |
| FGA | 73978329 | 6 | fibrinogen alpha chain [Canis lupus familiaris] | 0,400 | 0,222 |
| GAPDH | 1418260233 | 3 | glyceraldehyde-3-phosphate dehydrogenase-like [Canis lupus dingo] | 0,365 | 0,222 |
| GAPDH | 1418322993 | 3 | glyceraldehyde-3-phosphate dehydrogenase-like [Canis lupus dingo] | 0,365 | 0,222 |
| GAPDH | 1239896120 | 3 | glyceraldehyde-3-phosphate dehydrogenase-like [Canis lupus familiaris] | 0,365 | 0,222 |
| GAPDH | 1239963916 | 3 | glyceraldehyde-3-phosphate dehydrogenase-like [Canis lupus familiaris] | 0,365 | 0,222 |
| GSN | 1239925762 | 14 | gelsolin [Canis lupus familiaris] | 0,397 | 0,222 |
| GSN | 1418322411 | 14 | gelsolin isoform X1 [Canis lupus dingo] | 0,397 | 0,222 |
| GSN | 1418322413 | 14 | gelsolin isoform X2 [Canis lupus dingo] | 0,397 | 0,222 |
| GSN | 1418322415 | 14 | gelsolin isoform X3 [Canis lupus dingo] | 0,397 | 0,222 |
| HISTH4 | 50542205 | 2 | histone H4, partial [Canis lupus familiaris] | 0,763 | 0,222 |
| HISTH4 | 1418253529 | 5 | histone H4-like [Canis lupus dingo] | 0,775 | 0,222 |
| HP | 258498 | 2 | haptoglobin light chain, HpL chain [dogs, Peptide, 83 aa] | 0,752 | 0,222 |
| HSPA2 | 57090217 | 9 | heat shock-related 70 kDa protein 2 [Canis lupus familiaris] | 0,750 | 0,222 |
| HSPA8 | 545497049 | 11 | heat shock cognate 71 kDa protein [Canis lupus familiaris] | 0,561 | 0,222 |
| PDIA3 | 73964749 | 15 | protein disulfide-isomerase [Canis lupus familiaris] | 0,354 | 0,222 |
| PDIA3 | 1418259105 | 14 | protein disulfide-isomerase A3 [Canis lupus dingo] | 0,455 | 0,222 |
| PFN1 | 1418313130 | 6 | profilin-1 [Canis lupus dingo] | 0,572 | 0,222 |
| PPIA | 8699209 | 5 | cyclophilin A, partial [Canis lupus familiaris] | 0,457 | 0,222 |
| PPIA | 1239883019 | 3 | peptidyl-prolyl cis-trans isomerase A [Canis lupus familiaris] | 0,590 | 0,222 |
| PPIA | 1418202784 | 4 | peptidyl-prolyl cis-trans isomerase A isoform X1 [Canis lupus dingo] | 0,457 | 0,222 |
| PPIB | 1418257887 | 6 | peptidyl-prolyl cis-trans isomerase B [Canis lupus dingo] | 0,577 | 0,222 |
| PRDX4 | 1239984522 | 2 | peroxiredoxin-4 isoform X1 [Canis lupus familiaris] | 0,380 | 0,222 |
| PRDX4 | 1418203900 | 2 | peroxiredoxin-4 isoform X2 [Canis lupus dingo] | 0,380 | 0,222 |
| PRDX4 | 545557700 | 2 | peroxiredoxin-4 isoform X3 [Canis lupus familiaris] | 0,380 | 0,222 |
| PRDX4 | 1239984526 | 2 | peroxiredoxin-4 isoform X4 [Canis lupus familiaris] | 0,380 | 0,222 |
| PRDX4 | 1418203906 | 2 | peroxiredoxin-4 isoform X5 [Canis lupus dingo] | 0,380 | 0,222 |
| PRDX4 | 359324135 | 2 | peroxiredoxin-4 isoform X6 [Canis lupus familiaris] | 0,380 | 0,222 |
| PRDX4 | 359324139 | 2 | peroxiredoxin-4 isoform X7 [Canis lupus familiaris] | 0,380 | 0,222 |
| S100A6 | 1239912330 | 4 | protein S100-A6 [Canis lupus familiaris] | 0,667 | 0,222 |
| TAGLN2 | 345797882 | 3 | transgelin-2 [Canis lupus familiaris] | 0,291 | 0,222 |
| TF | 1418510423 | 2 | serotransferrin-like [Canis lupus dingo] | 0,405 | 0,222 |
| VDB | 1418328657 | 9 | vitamin D-binding protein [Canis lupus dingo] | 0,238 | 0,222 |
| LPO | 1418338512 | 26 | lactoperoxidase [Canis lupus dingo] | -0,983 | 0,240 |
| LPO | 1239919186 | 24 | lactoperoxidase [Canis lupus familiaris] | -0,990 | 0,240 |
| BPIFB2 | 1418249549 | 11 | BPI fold-containing family B member 2 [Canis lupus dingo] | -0,349 | 0,247 |
| ANXA1 | 558695394 | 7 | annexin A1 [Canis lupus familiaris] | 0,406 | 0,286 |
| CP | 1418510222 | 9 | ceruloplasmin isoform X1 [Canis lupus dingo] | 0,285 | 0,286 |
| CP | 1418510226 | 9 | ceruloplasmin isoform X2 [Canis lupus dingo] | 0,285 | 0,286 |
| CP | 1418510228 | 9 | ceruloplasmin isoform X3 [Canis lupus dingo] | 0,285 | 0,286 |
| CP | 1418510230 | 9 | ceruloplasmin isoform X4 [Canis lupus dingo] | 0,285 | 0,286 |
| CSTM | 1418503443 | 4 | cystatin-M [Canis lupus dingo] | -0,140 | 0,286 |
| HSP90B1 | 50979166 | 6 | endoplasmin precursor [Canis lupus familiaris] | 0,525 | 0,286 |
| HSP90B1 | 672890024 | 6 | heat shock protein 90 kDa beta member 1, partial [Canis lupus familiaris] | 0,525 | 0,286 |
| HSP90B1 | 159794957 | 5 | Chain B, Endoplasmin | 0,525 | 0,286 |
| HSP90B1 | 159794959 | 5 | Chain B, Endoplasmin | 0,525 | 0,286 |
| HSP90B1 | 1258500452 | 5 | Chain B, Endoplasmin | 0,525 | 0,286 |
| IGH-CH2 | 124390009 | 2 | immunoglobulin heavy chain constant region CH2, partial [Canis lupus familiaris] | 0,468 | 0,286 |
| PGD | 1418514978 | 11 | 6-phosphogluconate dehydrogenase, decarboxylating [Canis lupus dingo] | 0,403 | 0,286 |
| A2ML1 | 1418213939 | 18 | alpha-2-macroglobulin-like protein 1 isoform X2 [Canis lupus dingo] | 0,376 | 0,310 |
| A2ML1 | 1239967622 | 18 | alpha-2-macroglobulin-like protein 1 isoform X2 [Canis lupus familiaris] | 0,376 | 0,310 |
| A2ML1 | 1418213935 | 21 | alpha-2-macroglobulin-like protein 1 isoform X1 [Canis lupus dingo] | 0,387 | 0,310 |
| A2ML1 | 1239967618 | 21 | alpha-2-macroglobulin-like protein 1 isoform X1 [Canis lupus familiaris] | 0,387 | 0,310 |
| AMY2A | 1418288646 | 16 | pancreatic alpha-amylase [Canis lupus dingo] | 0,361 | 0,310 |
| AMY2A | 74013104 | 16 | pancreatic alpha-amylase [Canis lupus familiaris] | 0,361 | 0,310 |
| CALR | 1418219369 | 8 | calreticulin [Canis lupus dingo] | 0,483 | 0,310 |
| CALR | 345787749 | 8 | calreticulin [Canis lupus familiaris] | 0,483 | 0,310 |
| CP | 928167527 | 8 | ceruloplasmin isoform X1 [Canis lupus familiaris] | 0,222 | 0,310 |
| CP | 1239957836 | 8 | ceruloplasmin isoform X2 [Canis lupus familiaris] | 0,222 | 0,310 |
| CP | 73990367 | 8 | ceruloplasmin isoform X3 [Canis lupus familiaris] | 0,222 | 0,310 |
| CP | 1239957839 | 8 | ceruloplasmin isoform X4 [Canis lupus familiaris] | 0,222 | 0,310 |
| FGA | 1304047 | 3 | fibrinogen A-alpha-chain, partial [Canis lupus familiaris] | 0,380 | 0,310 |
| GAPDH | 8918232 | 2 | glyceraldehyde-3-phosphate dehydrogenase, partial [Canis lupus familiaris] | 0,428 | 0,310 |
| GAPDH | 1239975750 | 3 | glyceraldehyde-3-phosphate dehydrogenase-like [Canis lupus familiaris] | 0,433 | 0,310 |
| GAPDH | 1418333961 | 2 | glyceraldehyde-3-phosphate dehydrogenase-like isoform X1 [Canis lupus dingo] | 0,428 | 0,310 |
| GAPDH | 1418243111 | 3 | glyceraldehyde-3-phosphate dehydrogenase-like isoform X1 [Canis lupus dingo] | 0,433 | 0,310 |
| GAPDH | 1418333963 | 2 | glyceraldehyde-3-phosphate dehydrogenase-like isoform X2 [Canis lupus dingo] | 0,428 | 0,310 |
| GAPDH | 1418243113 | 3 | glyceraldehyde-3-phosphate dehydrogenase-like isoform X2 [Canis lupus dingo] | 0,433 | 0,310 |
| GSTA4 | 1418292151 | 3 | glutathione S-transferase A4-like [Canis lupus dingo] | 0,266 | 0,310 |
| GSTA4 | 1239929974 | 3 | glutathione S-transferase A4-like [Canis lupus familiaris] | 0,266 | 0,310 |
| HSPA5 | 345806081 | 13 | 78 kDa glucose-regulated protein [Canis lupus familiaris] | 0,431 | 0,310 |
| HSPA5 | 1418264760 | 13 | endoplasmic reticulum chaperone BiP [Canis lupus dingo] | 0,431 | 0,310 |
| LMNA | 560891942 | 3 | lamin [Canis lupus familiaris] | 0,449 | 0,310 |
| NQO2 | 1418205354 | 3 | ribosyldihydronicotinamide dehydrogenase [quinone] [Canis lupus dingo] | 0,457 | 0,310 |
| NQO2 | 74003808 | 3 | ribosyldihydronicotinamide dehydrogenase [quinone] [Canis lupus familiaris] | 0,457 | 0,310 |
| S100A4 | 1391723726 | 3 | protein S100-A4 isoform 1 [Canis lupus familiaris] | 1,031 | 0,310 |
| S100A4 | 1473222592 | 3 | protein S100-A4 isoform 2 [Canis lupus familiaris] | 1,031 | 0,310 |
| SPINK5 | 1418507526 | 11 | serine protease inhibitor Kazal-type 5 [Canis lupus dingo] | 0,591 | 0,310 |
| SPINK5 | 70794744 | 11 | serine protease inhibitor Kazal-type 5 precursor [Canis lupus familiaris] | 0,591 | 0,310 |
| YWHAQ | 1418213116 | 7 | 14-3-3 protein theta [Canis lupus dingo] | 0,650 | 0,310 |
| ZG16B | 73959451 | 5 | zymogen granule protein 16 homolog B [Canis lupus familiaris] | -0,697 | 0,394 |
| N/A | 21538359 | 3 | unnamed protein product, partial [Canis lupus familiaris] | 1,113 | 0,400 |
| N/A | 1418251085 | 4 | vomeromodulin-like [Canis lupus dingo] | -0,340 | 0,400 |
| ACSS2 | 1239959692 | 2 | acetyl-coenzyme A synthetase, cytoplasmic isoform X1 [Canis lupus familiaris] | 0,837 | 0,400 |
| ACSS2 | 1418251268 | 2 | acetyl-coenzyme A synthetase, cytoplasmic isoform X2 [Canis lupus dingo] | 0,837 | 0,400 |
| ACSS2 | 1239959695 | 2 | acetyl-coenzyme A synthetase, cytoplasmic isoform X3 [Canis lupus familiaris] | 0,837 | 0,400 |
| AHNAK | 1418504472 | 2 | neuroblast differentiation-associated protein AHNAK isoform X1 [Canis lupus dingo] | 0,397 | 0,400 |
| AHNAK | 928160186 | 2 | neuroblast differentiation-associated protein AHNAK isoform X1 [Canis lupus familiaris] | 0,397 | 0,400 |
| AHNAK | 1418504474 | 2 | neuroblast differentiation-associated protein AHNAK isoform X2 [Canis lupus dingo] | 0,397 | 0,400 |
| AHNAK | 1239946887 | 2 | neuroblast differentiation-associated protein AHNAK isoform X2 [Canis lupus familiaris] | 0,397 | 0,400 |
| AHNAK | 1418504476 | 2 | neuroblast differentiation-associated protein AHNAK isoform X3 [Canis lupus dingo] | 0,397 | 0,400 |
| AHNAK | 928160188 | 2 | neuroblast differentiation-associated protein AHNAK isoform X3 [Canis lupus familiaris] | 0,397 | 0,400 |
| AHNAK | 1418504478 | 2 | neuroblast differentiation-associated protein AHNAK isoform X4 [Canis lupus dingo] | 0,397 | 0,400 |
| ARPC3 | 1239964305 | 2 | actin-related protein 2/3 complex subunit 3 isoform X1 [Canis lupus familiaris] | 0,951 | 0,400 |
| ARPC4 | 1418261548 | 2 | actin-related protein 2/3 complex subunit 3 isoform X2 [Canis lupus dingo] | 0,951 | 0,400 |
| ARPC5 | 1418261550 | 2 | actin-related protein 2/3 complex subunit 3 isoform X3 [Canis lupus dingo] | 0,951 | 0,400 |
| ARSF | 1418203142 | 2 | arylsulfatase F isoform X1 [Canis lupus dingo] | -0,398 | 0,400 |
| ARSF | 1418203154 | 2 | arylsulfatase F isoform X2 [Canis lupus dingo] | -0,398 | 0,400 |
| ARSF | 1418203156 | 2 | arylsulfatase F isoform X3 [Canis lupus dingo] | -0,398 | 0,400 |
| ARSF | 81158058 | 2 | TPA: arylsulfatase F, partial [Canis lupus familiaris] | -0,398 | 0,400 |
| CTRC | 1418506315 | 7 | chymotrypsin-C-like [Canis lupus dingo] | -0,398 | 0,400 |
| EFHD2 | 1418506694 | 2 | EF-hand domain-containing protein D2 [Canis lupus dingo] | 0,653 | 0,400 |
| GDI1 | 4103761 | 3 | GDP dissociation inhibitor isoform 1 [Canis lupus familiaris] | 0,531 | 0,400 |
| GDI1 | 1418225109 | 3 | rab GDP dissociation inhibitor alpha [Canis lupus dingo] | 0,531 | 0,400 |
| GLO1 | 345778725 | 2 | lactoylglutathione lyase [Canis lupus familiaris] | 0,472 | 0,400 |
| LAP3 | 545493958 | 12 | cytosol aminopeptidase [Canis lupus familiaris] | 0,316 | 0,400 |
| PIR | 1418203467 | 2 | pirin isoform X1 [Canis lupus dingo] | 1,136 | 0,400 |
| PIR | 1239984629 | 2 | pirin isoform X2 [Canis lupus familiaris] | 1,136 | 0,400 |
| PKM | 1239972768 | 10 | pyruvate kinase PKM isoform X1 [Canis lupus familiaris] | 0,423 | 0,400 |
| PKM | 1418257040 | 9 | pyruvate kinase PKM isoform X2 [Canis lupus dingo] | 0,442 | 0,400 |
| PKM | 1418257042 | 10 | pyruvate kinase PKM isoform X3 [Canis lupus dingo] | 0,423 | 0,400 |
| PKM | 545550333 | 9 | pyruvate kinase PKM isoform X3 [Canis lupus familiaris] | 0,442 | 0,400 |
| PKM | 1418257044 | 8 | pyruvate kinase PKM isoform X4 [Canis lupus dingo] | 0,423 | 0,400 |
| UBA1 | 928183828 | 4 | LOW QUALITY PROTEIN: ubiquitin-like modifier-activating enzyme 1 [Canis lupus familiaris] | 0,548 | 0,400 |
| UBA1 | 1418249227 | 4 | ubiquitin-like modifier-activating enzyme 1 [Canis lupus dingo] | 0,548 | 0,400 |
| UBE1Y | 823758757 | 2 | UBE1Y [Canis lupus familiaris] | 0,548 | 0,400 |
| UBE1Y | 528889162 | 2 | UBE1Y transcript 1 [Canis lupus familiaris] | 0,548 | 0,400 |
| UBE1Y | 528889178 | 2 | UBE1Y transcript 2 [Canis lupus familiaris] | 0,548 | 0,400 |
| UBE1Y | 528889180 | 2 | UBE1Y transcript 3 [Canis lupus familiaris] | 0,548 | 0,400 |
| LCP1 | 1239955970 | 17 | plastin-2 isoform X1 [Canis lupus familiaris] | 0,229 | 0,413 |
| LCP1 | 545537421 | 17 | plastin-2 isoform X2 [Canis lupus familiaris] | 0,229 | 0,413 |
| MUC7 | 1418328462 | 3 | mucin-7 [Canis lupus dingo] | 0,463 | 0,413 |
| MUC7 | 1239883384 | 3 | mucin-7-like [Canis lupus familiaris] | 0,463 | 0,413 |
| ADIRF | 1239898218 | 3 | adipogenesis regulatory factor [Canis lupus familiaris] | 0,573 | 0,421 |
| CALM2 | 1418197013 | 2 | calmodulin-2 [Canis lupus dingo] | 0,098 | 0,421 |
| GAPDH | 6983847 | 5 | glyceraldehyde-3-phosphate dehydrogenase [Canis lupus familiaris] | 0,321 | 0,421 |
| GAPDH | 925115133 | 5 | glyceraldehyde-3-phosphate dehydrogenase [Canis lupus familiaris] | 0,321 | 0,421 |
| GAPDH | 1418255985 | 4 | glyceraldehyde-3-phosphate dehydrogenase isoform X1 [Canis lupus dingo] | 0,321 | 0,421 |
| GAPDH | 1418292394 | 5 | glyceraldehyde-3-phosphate dehydrogenase isoform X1 [Canis lupus dingo] | 0,321 | 0,421 |
| GAPDH | 1418255987 | 4 | glyceraldehyde-3-phosphate dehydrogenase isoform X2 [Canis lupus dingo] | 0,321 | 0,421 |
| GAPDH | 1418292396 | 5 | glyceraldehyde-3-phosphate dehydrogenase isoform X2 [Canis lupus dingo] | 0,321 | 0,421 |
| GAPDH | 1418222910 | 4 | glyceraldehyde-3-phosphate dehydrogenase-like isoform X1 [Canis lupus dingo] | 0,321 | 0,421 |
| GAPDH | 1418243839 | 5 | glyceraldehyde-3-phosphate dehydrogenase-like isoform X1 [Canis lupus dingo] | 0,321 | 0,421 |
| GAPDH | 1418333597 | 4 | glyceraldehyde-3-phosphate dehydrogenase-like isoform X1 [Canis lupus dingo] | 0,321 | 0,421 |
| GAPDH | 1418222926 | 4 | glyceraldehyde-3-phosphate dehydrogenase-like isoform X2 [Canis lupus dingo] | 0,321 | 0,421 |
| GAPDH | 1418243841 | 5 | glyceraldehyde-3-phosphate dehydrogenase-like isoform X2 [Canis lupus dingo] | 0,321 | 0,421 |
| GAPDH | 1418333599 | 4 | glyceraldehyde-3-phosphate dehydrogenase-like isoform X2 [Canis lupus dingo] | 0,321 | 0,421 |
| GDI2 | 1418501820 | 4 | rab GDP dissociation inhibitor beta isoform X1 [Canis lupus dingo] | 0,219 | 0,421 |
| GDI2 | 1418501822 | 4 | rab GDP dissociation inhibitor beta isoform X2 [Canis lupus dingo] | 0,219 | 0,421 |
| GDI2 | 1418501824 | 4 | rab GDP dissociation inhibitor beta isoform X3 [Canis lupus dingo] | 0,219 | 0,421 |
| GSTM1 | 1418288349 | 10 | glutathione S-transferase Mu 1 isoform X1 [Canis lupus dingo] | 0,388 | 0,421 |
| GSTM1 | 1239908416 | 9 | glutathione S-transferase Mu 1 isoform X2 [Canis lupus familiaris] | 0,393 | 0,421 |
| HSP90AA1 | 928142969 | 5 | heat shock protein HSP 90-alpha [Canis lupus familiaris] | 0,439 | 0,421 |
| HYAL1 | 545533633 | 10 | hyaluronidase-1 isoform X1 [Canis lupus familiaris] | -0,243 | 0,421 |
| HYAL1 | 1418215838 | 10 | hyaluronidase-1 isoform X2 [Canis lupus dingo] | -0,243 | 0,421 |
| HYAL1 | 1239950142 | 8 | hyaluronidase-1 isoform X3 [Canis lupus familiaris] | -0,215 | 0,421 |
| LOC106559694 | 928153482 | 4 | LOW QUALITY PROTEIN: glyceraldehyde-3-phosphate dehydrogenase-like [Canis lupus familiaris] | 0,317 | 0,421 |
| NCCRP1 | 73948372 | 5 | F-box only protein 50 [Canis lupus familiaris] | 0,230 | 0,421 |
| PRDX1 | 1418294943 | 7 | peroxiredoxin-1 [Canis lupus dingo] | 0,227 | 0,421 |
| SBSN | 545488886 | 4 | suprabasin [Canis lupus familiaris] | 0,188 | 0,421 |
| SBSN | 1418297849 | 4 | suprabasin isoform X1 [Canis lupus dingo] | 0,188 | 0,421 |
| SBSN | 1418297851 | 4 | suprabasin isoform X2 [Canis lupus dingo] | 0,188 | 0,421 |
| XDH | 1418212080 | 16 | xanthine dehydrogenase/oxidase isoform X1 [Canis lupus dingo] | 0,374 | 0,421 |
| XDH | 73980076 | 16 | xanthine dehydrogenase/oxidase isoform X1 [Canis lupus familiaris] | 0,374 | 0,421 |
| XDH | 1418212082 | 16 | xanthine dehydrogenase/oxidase isoform X2 [Canis lupus dingo] | 0,374 | 0,421 |
| XDH | 545527502 | 16 | xanthine dehydrogenase/oxidase isoform X2 [Canis lupus familiaris] | 0,374 | 0,421 |
| YWHAE | 1418267412 | 8 | 14-3-3 protein epsilon isoform X1 [Canis lupus dingo] | 0,431 | 0,421 |
| ACTA1 | 1418509163 | 2 | actin, alpha skeletal muscle [Canis lupus dingo] | 0,091 | 0,429 |
| ACTA2 | 1418341514 | 2 | actin, aortic smooth muscle [Canis lupus dingo] | 0,091 | 0,429 |
| ACTC1 | 57108093 | 2 | actin, alpha cardiac muscle 1 [Canis lupus familiaris] | 0,091 | 0,429 |
| ACTG2 | 1418210682 | 2 | actin, gamma-enteric smooth muscle [Canis lupus dingo] | 0,091 | 0,429 |
| PIP | 73978762 | 12 | prolactin-inducible protein [Canis lupus familiaris] | -0,295 | 0,429 |
| IGJ; JCHAIN | 345779666 | 10 | immunoglobulin J chain [Canis lupus familiaris] | 0,131 | 0,457 |
| UBB | 1239889921 | 3 | ubiquitin-like [Canis lupus familiaris] | 0,202 | 0,485 |
| UBB | 1239954579 | 4 | ubiquitin-like, partial [Canis lupus familiaris] | 0,206 | 0,485 |
| PNP | 1418335936 | 6 | purine nucleoside phosphorylase [Canis lupus dingo] | 0,039 | 0,486 |
| N/A | 1239959951 | 6 | vomeromodulin-like [Canis lupus familiaris] | -0,354 | 0,500 |
| APOH | 296089 | 2 | apolipoprotein H; beta-2-glycoprotein I [Canis lupus familiaris] | 0,217 | 0,548 |
| AZGP1 | 70909945 | 7 | zinc alpha-2-glycoprotein 1, partial [Canis lupus familiaris] | -0,143 | 0,548 |
| AZGP1 | 560879429 | 8 | zinc-alpha-2-glycoprotein precursor [Canis lupus familiaris] | -0,143 | 0,548 |
| CD44 | 1418208383 | 2 | CD44 antigen isoform X10 [Canis lupus dingo] | 0,369 | 0,548 |
| CD44 | 1418208367 | 2 | CD44 antigen isoform X2 [Canis lupus dingo] | 0,369 | 0,548 |
| CD44 | 545529575 | 2 | CD44 antigen isoform X2 [Canis lupus familiaris] | 0,369 | 0,548 |
| CD44 | 1418208369 | 2 | CD44 antigen isoform X3 [Canis lupus dingo] | 0,369 | 0,548 |
| CD44 | 1418208371 | 2 | CD44 antigen isoform X4 [Canis lupus dingo] | 0,369 | 0,548 |
| CD44 | 1418208373 | 2 | CD44 antigen isoform X5 [Canis lupus dingo] | 0,369 | 0,548 |
| CD44 | 545529581 | 2 | CD44 antigen isoform X6 [Canis lupus familiaris] | 0,369 | 0,548 |
| CD44 | 1239944036 | 2 | CD44 antigen isoform X7 [Canis lupus familiaris] | 0,369 | 0,548 |
| CD44 | 545529583 | 2 | CD44 antigen isoform X8 [Canis lupus familiaris] | 0,369 | 0,548 |
| CD44 | 1239944039 | 2 | CD44 antigen isoform X9 [Canis lupus familiaris] | 0,369 | 0,548 |
| CES1 | 1418507047 | 7 | liver carboxylesterase 1 [Canis lupus dingo] | -0,166 | 0,548 |
| CLU | 163954 | 7 | glycoprotein 80 [Canis lupus familiaris] | 0,394 | 0,548 |
| CORO1A | 1418213540 | 7 | cornifin-A [Canis lupus dingo] | 0,406 | 0,548 |
| GPI | 1418305580 | 18 | LOW QUALITY PROTEIN: glucose-6-phosphate isomerase [Canis lupus dingo] | 0,209 | 0,548 |
| HSPB1 | 696633650 | 4 | heat shock protein 27, partial [Canis lupus familiaris] | 0,315 | 0,548 |
| HSPB1 | 1418307159 | 5 | heat shock protein beta-1 [Canis lupus dingo] | 0,252 | 0,548 |
| HSPB1 | 924442944 | 5 | heat shock protein beta-1 [Canis lupus familiaris] | 0,252 | 0,548 |
| HSPB1 | 624685 | 5 | heat-shock protein [Canis lupus familiaris] | 0,252 | 0,548 |
| IVL | 545528998 | 2 | involucrin [Canis lupus familiaris] | 0,545 | 0,548 |
| PEBP1 | 114326321 | 5 | phosphatidylethanolamine-binding protein 1 [Canis lupus familiaris] | 0,154 | 0,548 |
| S100A11 | 928158750 | 3 | protein S100-A11 [Canis lupus familiaris] | 0,430 | 0,548 |
| S100A11 | 1418213478 | 3 | protein S100-A11, partial [Canis lupus dingo] | 0,430 | 0,548 |
| SERPINA1 | 119637732 | 5 | alpha 1 antitrypsin [Canis lupus familiaris] | 0,102 | 0,548 |
| SERPINA1 | 1239913623 | 5 | alpha-1-antitrypsin isoform X1 [Canis lupus familiaris] | 0,102 | 0,548 |
| SERPINA1 | 1418345511 | 5 | alpha-1-antitrypsin-like [Canis lupus dingo] | 0,102 | 0,548 |
| SERPINB5 | 1418298970 | 5 | serpin B5 isoform X1 [Canis lupus dingo] | 0,413 | 0,548 |
| SERPINB5 | 345784333 | 5 | serpin B5 isoform X1 [Canis lupus familiaris] | 0,413 | 0,548 |
| SERPINB5 | 1418298974 | 5 | serpin B5 isoform X2 [Canis lupus dingo] | 0,413 | 0,548 |
| SERPINB5 | 1239884495 | 5 | serpin B5 isoform X2 [Canis lupus familiaris] | 0,413 | 0,548 |
| SLPI | 164499359 | 4 | secretory leukocyte peptidase inhibitor, partial [Canis lupus familiaris] | -0,278 | 0,548 |
| SOD1 | 18150346 | 5 | Cu/Zn superoxide dismutase [Canis lupus familiaris] | 0,032 | 0,548 |
| TPI1 | 76363530 | 14 | RecName: Full=Triosephosphate isomerase; Short=TIM; AltName: Full=Methylglyoxal synthase; AltName: Full=Triose-phosphate isomerase | 0,195 | 0,548 |
| TPM3 | 545504091 | 3 | tropomyosin alpha-3 chain isoform X6 [Canis lupus familiaris] | 0,433 | 0,548 |
| TPM3 | 1418313468 | 3 | tropomyosin alpha-3 chain isoform X7 [Canis lupus dingo] | 0,433 | 0,548 |
| YWHAE | 73967156 | 9 | 14-3-3 protein epsilon isoform X2 [Canis lupus familiaris] | 0,398 | 0,548 |
| YWHAE | 1418267416 | 6 | 14-3-3 protein epsilon isoform X3 [Canis lupus dingo] | 0,431 | 0,548 |
| YWHAE | 1418267418 | 7 | 14-3-3 protein epsilon isoform X4 [Canis lupus dingo] | 0,448 | 0,548 |
| PGK1 | 74007807 | 7 | phosphoglycerate kinase 1 [Canis lupus familiaris] | 0,381 | 0,556 |
| IGJ; JCHAIN | 19715661 | 5 | immunoglobulin J chain, partial [Canis lupus familiaris] | 0,353 | 0,589 |
| S100P | 345798353 | 2 | protein S100-P [Canis lupus familiaris] | 0,087 | 0,589 |
| TF | 928167632 | 3 | serotransferrin [Canis lupus familiaris] | 0,169 | 0,589 |
| UBA52 | 1418311328 | 5 | ubiquitin [Canis lupus dingo] | 0,206 | 0,589 |
| UBA52 | 5822852 | 6 | ubiquitin, partial [Canis lupus familiaris] | 0,206 | 0,589 |
| UBA52 | 356582340 | 6 | ubiquitin-40S ribosomal protein S27a [Canis lupus familiaris] | 0,206 | 0,589 |
| UBA52 | 5441519 | 6 | ubiquitin-ribosomal protein L40 fusion protein [Canis lupus familiaris] | 0,206 | 0,589 |
| UBB | 1418312167 | 6 | polyubiquitin-B [Canis lupus dingo] | 0,206 | 0,589 |
| UBB | 1239902229 | 6 | polyubiquitin-B [Canis lupus familiaris] | 0,206 | 0,589 |
| UBC | 1418260846 | 6 | polyubiquitin-C [Canis lupus dingo] | 0,206 | 0,589 |
| UBC | 73995130 | 6 | polyubiquitin-C [Canis lupus familiaris] | 0,206 | 0,589 |
| BPIFA2 | 1418249553 | 16 | BPI fold-containing family A member 2 [Canis lupus dingo] | 1,056 | 0,662 |
| KRT4 | 1418221457 | 17 | keratin, type II cytoskeletal 4 [Canis lupus dingo] | 0,041 | 0,662 |
| PRSS2 | 1418324407 | 6 | anionic trypsin [Canis lupus dingo] | 0,175 | 0,662 |
| PRSS2 | 116583308 | 6 | anionic trypsinogen, partial [Canis lupus familiaris] | 0,175 | 0,662 |
| HSP90AA1 | 1418344784 | 6 | heat shock protein HSP 90-alpha [Canis lupus dingo] | 0,517 | 0,683 |
| N/A | 1239884102 | 5 | phospholipase A2 inhibitor and Ly6/PLAUR domain-containing protein, partial [Canis lupus familiaris] | -0,430 | 0,690 |
| N/A | 1418192575 | 11 | submaxillary mucin-like protein, partial [Canis lupus dingo] | -0,080 | 0,690 |
| N/A | 16607724 | 5 | unnamed protein product [Canis lupus familiaris] | -0,291 | 0,690 |
| N/A | 16607681 | 5 | unnamed protein product, partial [Canis lupus familiaris] | -0,291 | 0,690 |
| CALM3 | 1418304395 | 2 | calmodulin-3 isoform X2 [Canis lupus dingo] | -0,031 | 0,690 |
| CDH1 | 1418308718 | 8 | cadherin-1 [Canis lupus dingo] | 0,076 | 0,690 |
| GAPDH | 1418256458 | 2 | glyceraldehyde-3-phosphate dehydrogenase-like [Canis lupus dingo] | 0,120 | 0,690 |
| GAPDH | 928176932 | 2 | glyceraldehyde-3-phosphate dehydrogenase-like [Canis lupus familiaris] | 0,120 | 0,690 |
| IGH-CH4 | 124390013 | 2 | immunoglobulin heavy chain constant region CH4, partial [Canis lupus familiaris] | 0,437 | 0,690 |
| IGHGD | 17066530 | 5 | immunoglobulin gamma heavy chain D [Canis lupus familiaris] | -0,291 | 0,690 |
| IGM | 146743249 | 2 | immunoglobulin mu heavy chain variable region, partial [Canis lupus familiaris] | 0,437 | 0,690 |
| LOC611458 | 1418213976 | 5 | pregnancy zone protein-like isoform X1 [Canis lupus dingo] | 0,104 | 0,690 |
| LOC611458 | 545546412 | 5 | pregnancy zone protein-like isoform X1 [Canis lupus familiaris] | 0,104 | 0,690 |
| LOC611458 | 1418213978 | 5 | pregnancy zone protein-like isoform X2 [Canis lupus dingo] | 0,104 | 0,690 |
| LOC611458 | 545546414 | 5 | pregnancy zone protein-like isoform X2 [Canis lupus familiaris] | 0,104 | 0,690 |
| LOC611458 | 1418213980 | 5 | pregnancy zone protein-like isoform X3 [Canis lupus dingo] | 0,104 | 0,690 |
| LOC611458 | 1239967247 | 5 | pregnancy zone protein-like isoform X3 [Canis lupus familiaris] | 0,104 | 0,690 |
| MPO | 545511447 | 7 | myeloperoxidase [Canis lupus familiaris] | 0,159 | 0,690 |
| NPC2 | 945179 | 3 | CE1 [Canis lupus familiaris] | 0,103 | 0,690 |
| NPC2 | 356582247 | 3 | NPC intracellular cholesterol transporter 2 precursor [Canis lupus familiaris] | 0,103 | 0,690 |
| PEBP4 | 1418230432 | 3 | phosphatidylethanolamine-binding protein 4 isoform X1 [Canis lupus dingo] | -0,185 | 0,690 |
| PEBP4 | 345790561 | 2 | phosphatidylethanolamine-binding protein 4 isoform X1 [Canis lupus familiaris] | -0,270 | 0,690 |
| PEBP4 | 1418230422 | 3 | phosphatidylethanolamine-binding protein 4 isoform X2 [Canis lupus dingo] | -0,185 | 0,690 |
| PEBP4 | 1239961995 | 2 | phosphatidylethanolamine-binding protein 4 isoform X2 [Canis lupus familiaris] | -0,270 | 0,690 |
| PEBP4 | 1418230426 | 3 | phosphatidylethanolamine-binding protein 4 isoform X4 [Canis lupus dingo] | -0,185 | 0,690 |
| PEBP4 | 1239961999 | 2 | phosphatidylethanolamine-binding protein 4 isoform X4 [Canis lupus familiaris] | -0,270 | 0,690 |
| PEBP4 | 1418230428 | 3 | phosphatidylethanolamine-binding protein 4 isoform X5 [Canis lupus dingo] | -0,185 | 0,690 |
| PEBP4 | 1239962001 | 2 | phosphatidylethanolamine-binding protein 4 isoform X5 [Canis lupus familiaris] | -0,270 | 0,690 |
| PEBP4 | 1418230430 | 3 | phosphatidylethanolamine-binding protein 4 isoform X6 [Canis lupus dingo] | -0,185 | 0,690 |
| PEBP4 | 345790559 | 2 | phosphatidylethanolamine-binding protein 4 isoform X6 [Canis lupus familiaris] | -0,270 | 0,690 |
| PGAM1 | 345792633 | 6 | phosphoglycerate mutase 1 [Canis lupus familiaris] | 0,145 | 0,690 |
| RHOA | 545533091 | 2 | transforming protein RhoA isoform X1 [Canis lupus familiaris] | 0,329 | 0,690 |
| SLPI | 158936956 | 5 | protease inhibitor [Canis lupus familiaris] | -0,209 | 0,690 |
| TFF1 | 30144609 | 2 | trefoil factor 1 [Canis lupus familiaris] | -0,670 | 0,690 |
| TPM3 | 545504087 | 2 | tropomyosin alpha-3 chain isoform X7 [Canis lupus familiaris] | 0,331 | 0,690 |
| TPM3 | 1418313472 | 2 | tropomyosin alpha-3 chain isoform X9 [Canis lupus dingo] | 0,331 | 0,690 |
| TPM4 | 73986126 | 2 | tropomyosin alpha-4 chain isoform X1 [Canis lupus familiaris] | 0,211 | 0,690 |
| TPM4 | 1418219677 | 2 | tropomyosin alpha-4 chain isoform X3 [Canis lupus dingo] | 0,211 | 0,690 |
| YWHAG | 1418307161 | 4 | 14-3-3 protein gamma [Canis lupus dingo] | 0,317 | 0,690 |
| YWHAG | 1239905645 | 3 | 14-3-3 protein gamma [Canis lupus familiaris] | 0,317 | 0,690 |
| N/A | 60734607 | 9 | unnamed protein product, partial [Canis lupus familiaris] | 0,054 | 0,699 |
| MUC5B | 1418206782 | 13 | LOW QUALITY PROTEIN: mucin-5B [Canis lupus dingo] | -0,943 | 0,699 |
| SMR3A | 1239933313 | 7 | submaxillary gland androgen-regulated protein 3A isoform X1 [Canis lupus familiaris] | -0,377 | 0,699 |
| SMR3A | 345779658 | 7 | submaxillary gland androgen-regulated protein 3A isoform X2 [Canis lupus familiaris] | -0,377 | 0,699 |
| N/A | 1418213904 | 2 | histone H2B type 1-F/J/L-like [Canis lupus dingo] | 0,326 | 0,730 |
| N/A | 1239979162 | 2 | late histone H2B.L4 [Canis lupus familiaris] | 0,326 | 0,730 |
| N/A | 207008756 | 3 | unnamed protein product [Canis lupus familiaris] | 0,326 | 0,730 |
| G6PD | 1418225081 | 11 | glucose-6-phosphate 1-dehydrogenase isoform X1 [Canis lupus dingo] | -0,010 | 0,730 |
| G6PD | 1418225083 | 11 | glucose-6-phosphate 1-dehydrogenase isoform X2 [Canis lupus dingo] | -0,010 | 0,730 |
| HIST1H2B | 1418253565 | 3 | histone H2B type 1 [Canis lupus dingo] | 0,326 | 0,730 |
| HIST1H2B | 359324332 | 3 | histone H2B type 1-like, partial [Canis lupus familiaris] | 0,326 | 0,730 |
| HIST1H2BA | 74004168 | 3 | histone H2B type 1-A [Canis lupus familiaris] | 0,326 | 0,730 |
| HIST1H2BA | 1239979401 | 2 | histone H2B type 1-A [Canis lupus familiaris] | 0,326 | 0,730 |
| HIST1H2BB | 74004182 | 2 | histone H2B type 1-B [Canis lupus familiaris] | 0,326 | 0,730 |
| HIST1H2BJ | 1418253479 | 2 | histone H2B type 1-J [Canis lupus dingo] | 0,326 | 0,730 |
| HIST1H2BK | 1418253472 | 3 | histone H2B type 1-K [Canis lupus dingo] | 0,326 | 0,730 |
| HIST1H2BM | 1418204463 | 3 | histone H2B type 1-M [Canis lupus dingo] | 0,326 | 0,730 |
| HIST2H2BE | 545528721 | 2 | histone H2B type 2-E [Canis lupus familiaris] | 0,326 | 0,730 |
| HIST2H2BE | 1418253515 | 2 | histone H2B type 2-E-like [Canis lupus dingo] | 0,326 | 0,730 |
| HIST2H2BE | 545554810 | 2 | histone H2B type 2-E-like [Canis lupus familiaris] | 0,326 | 0,730 |
| HIST2H2BF | 1418209857 | 3 | histone H2B type 2-F [Canis lupus dingo] | 0,326 | 0,730 |
| HIST3H2BB | 1418213848 | 2 | histone H2B type 3-B [Canis lupus dingo] | 0,326 | 0,730 |
| IGH-CH1 | 124390007 | 3 | immunoglobulin heavy chain constant region CH1, partial [Canis lupus familiaris] | 0,746 | 0,730 |
| KRT5 | 1418221469 | 8 | keratin, type II cytoskeletal 5 [Canis lupus dingo] | -0,695 | 0,730 |
| KRT5 | 1069415302 | 8 | keratin, type II cytoskeletal 5 [Canis lupus familiaris] | -0,695 | 0,730 |
| LYZ | 9257149 | 3 | Chain A, X-Ray Crystal Structure Analysis Of Canine Milk Lysozyme (Apo-Type) | 0,291 | 0,730 |
| LYZ | 13787135 | 3 | Chain B, Lysozyme C | 0,291 | 0,730 |
| LYZ | 925114454 | 3 | lysozyme C, milk isozyme-like precursor [Canis lupus familiaris] | 0,291 | 0,730 |
| LYZ | 8928188 | 3 | RecName: Full=Lysozyme C, milk isozyme; AltName: Full=1,4-beta-N-acetylmuramidase C | 0,291 | 0,730 |
| SLPI | 1418249573 | 6 | antileukoproteinase-like [Canis lupus dingo] | 0,252 | 0,730 |
| N/A | 1418194692 | 2 | angiopoietin-related protein 5-like [Canis lupus dingo] | -0,108 | 0,792 |
| N/A | 1239891563 | 6 | double-headed protease inhibitor, submandibular gland [Canis lupus familiaris] | 0,240 | 0,792 |
| TXN | 1418321566 | 5 | thioredoxin [Canis lupus dingo] | 0,092 | 0,792 |
| TXN | 1239928271 | 5 | thioredoxin-like isoform X1 [Canis lupus familiaris] | 0,092 | 0,792 |
| TXN | 1239928273 | 5 | thioredoxin-like isoform X2 [Canis lupus familiaris] | 0,092 | 0,792 |
| N/A | 1418293613 | 5 | L-amino-acid oxidase-like [Canis lupus dingo] | 0,533 | 0,800 |
| BLVRB | 1418305165 | 2 | flavin reductase (NADPH) isoform X1 [Canis lupus dingo] | -0,163 | 0,800 |
| BLVRB | 1418305167 | 2 | flavin reductase (NADPH) isoform X2 [Canis lupus dingo] | -0,163 | 0,800 |
| C5 | 1418321303 | 2 | complement C5 [Canis lupus dingo] | 0,561 | 0,800 |
| C5 | 1239925760 | 2 | complement C5 [Canis lupus familiaris] | 0,561 | 0,800 |
| CLIC1 | 356460954 | 2 | chloride intracellular channel protein 1 [Canis lupus familiaris] | 0,525 | 0,800 |
| CTSG | 1418346371 | 4 | cathepsin G [Canis lupus dingo] | 0,297 | 0,800 |
| CTSG | 928141642 | 4 | cathepsin G [Canis lupus familiaris] | 0,297 | 0,800 |
| HSP90B1 | 39654740 | 3 | Chain A, Endoplasmin | 0,344 | 0,800 |
| HSP90B1 | 71042092 | 3 | Chain A, Endoplasmin | 0,344 | 0,800 |
| HSP90B1 | 1335512822 | 2 | Chain A, Endoplasmin | 0,344 | 0,800 |
| HSP90B1 | 1379069777 | 2 | Chain B, Endoplasmin | 0,344 | 0,800 |
| KRT14 | 1418338716 | 4 | keratin, type I cytoskeletal 14 [Canis lupus dingo] | -0,865 | 0,800 |
| MDH2 | 1418307145 | 5 | malate dehydrogenase, mitochondrial [Canis lupus dingo] | 0,505 | 0,800 |
| MDH2 | 1239955263 | 4 | malate dehydrogenase, mitochondrial-like [Canis lupus familiaris] | 0,505 | 0,800 |
| MDH2 | 89574135 | 4 | mitochondrial malate dehydrogenase 2, NAD, partial [Canis lupus familiaris] | 0,505 | 0,800 |
| MPO | 494395 | 3 | Chain C, Myeloperoxidase | 0,196 | 0,800 |
| MYL6 | 345776590 | 2 | myosin light polypeptide 6 isoform X1 [Canis lupus familiaris] | -0,119 | 0,800 |
| MYL6 | 1418269617 | 2 | myosin light polypeptide 6 isoform X2 [Canis lupus dingo] | -0,119 | 0,800 |
| MYL6 | 1239922056 | 2 | myosin light polypeptide 6 isoform X3 [Canis lupus familiaris] | -0,119 | 0,800 |
| PEPD | 1418305616 | 3 | xaa-Pro dipeptidase isoform X1 [Canis lupus dingo] | -0,341 | 0,800 |
| PEPD | 73948526 | 3 | xaa-Pro dipeptidase isoform X1 [Canis lupus familiaris] | -0,341 | 0,800 |
| PEPD | 545489037 | 2 | xaa-Pro dipeptidase isoform X2 [Canis lupus familiaris] | -0,341 | 0,800 |
| PLA2G7 | 1585652 | 4 | platelet-activating factor acetylhydrolase | -0,147 | 0,800 |
| PLA2G7 | 1418291961 | 4 | platelet-activating factor acetylhydrolase isoform X1 [Canis lupus dingo] | -0,147 | 0,800 |
| PLA2G7 | 545518207 | 4 | platelet-activating factor acetylhydrolase isoform X1 [Canis lupus familiaris] | -0,147 | 0,800 |
| PLA2G7 | 545518209 | 4 | platelet-activating factor acetylhydrolase isoform X2 [Canis lupus familiaris] | -0,147 | 0,800 |
| PLA2G7 | 1418291973 | 4 | platelet-activating factor acetylhydrolase isoform X3 [Canis lupus dingo] | -0,147 | 0,800 |
| PLG | 130314 | 3 | RecName: Full=Plasminogen; Contains: RecName: Full=Plasmin heavy chain A; Contains: RecName: Full=Plasmin light chain B | 0,086 | 0,800 |
| SERPING1 | 1418207941 | 2 | plasma protease C1 inhibitor [Canis lupus dingo] | 0,115 | 0,800 |
| TPT1 | 545537467 | 2 | translationally-controlled tumor protein [Canis lupus familiaris] | 0,029 | 0,800 |
| IGHAC | 598107 | 11 | IgA heavy chain constant region, partial [Canis lupus familiaris] | 0,177 | 0,818 |
| LOC611455 | 545546410 | 3 | ovostatin homolog 2-like [Canis lupus familiaris] | -0,072 | 0,818 |
| LYZ | 665505916 | 12 | lysozyme C precursor [Canis lupus familiaris] | -0,403 | 0,818 |
| LYZ | 8928189 | 12 | RecName: Full=Lysozyme C, spleen isozyme; AltName: Full=1,4-beta-N-acetylmuramidase C | -0,403 | 0,818 |
| S100A2 | 1418313584 | 6 | protein S100-A2 [Canis lupus dingo] | -0,082 | 0,818 |
| N/A | 645985805 | 2 | Chain C, Lipocalin Allergen | 0,047 | 0,841 |
| N/A | 1418287456 | 3 | serine protease 27 [Canis lupus dingo] | 0,164 | 0,841 |
| AHSG | 1418515495 | 4 | alpha-2-HS-glycoprotein [Canis lupus dingo] | 0,053 | 0,841 |
| AMY2A | 928138460 | 2 | pancreatic alpha-amylase [Canis lupus familiaris] | -0,089 | 0,841 |
| AMY2A | 928186395 | 11 | pancreatic alpha-amylase-like [Canis lupus familiaris] | -0,005 | 0,841 |
| APOA1 | 1418241889 | 18 | apolipoprotein A-I [Canis lupus dingo] | 0,117 | 0,841 |
| APOA1 | 3915607 | 17 | RecName: Full=Apolipoprotein A-I; Short=Apo-AI; Short=ApoA-I; AltName: Full=Apolipoprotein A1; Contains: RecName: Full=Proapolipoprotein A-I; Short=ProapoA-I; Contains: RecName: Full=Truncated apolipoprotein A-I; Flags: Precursor | 0,087 | 0,841 |
| BPIFA1 | 1418251076 | 3 | BPI fold-containing family A member 1 [Canis lupus dingo] | 0,251 | 0,841 |
| CALM1 | 1239893625 | 3 | calmodulin, partial [Canis lupus familiaris] | 0,049 | 0,841 |
| CAMP | 1418215404 | 6 | cathelicidin antimicrobial peptide [Canis lupus dingo] | 0,176 | 0,841 |
| CAMP | 50979214 | 7 | cathelicidin antimicrobial peptide precursor [Canis lupus familiaris] | 0,176 | 0,841 |
| ECM1 | 1418209738 | 2 | LOW QUALITY PROTEIN: extracellular matrix protein 1 [Canis lupus dingo] | -0,127 | 0,841 |
| ECM1 | 345782613 | 2 | LOW QUALITY PROTEIN: extracellular matrix protein 1 [Canis lupus familiaris] | -0,127 | 0,841 |
| GAPDH | 1418222735 | 2 | glyceraldehyde-3-phosphate dehydrogenase-like isoform X1 [Canis lupus dingo] | 0,130 | 0,841 |
| GAPDH | 1418222737 | 2 | glyceraldehyde-3-phosphate dehydrogenase-like isoform X2 [Canis lupus dingo] | 0,130 | 0,841 |
| HSPE1 | 1418202522 | 2 | 10 kDa heat shock protein, mitochondrial [Canis lupus dingo] | 0,110 | 0,841 |
| HSPE1 | 345797630 | 2 | 10 kDa heat shock protein, mitochondrial isoform X3 [Canis lupus familiaris] | 0,110 | 0,841 |
| KRT10 | 1418337563 | 8 | keratin, type I cytoskeletal 10 isoform X1 [Canis lupus dingo] | 0,171 | 0,841 |
| KRT10 | 928144438 | 8 | keratin, type I cytoskeletal 10 isoform X1 [Canis lupus familiaris] | 0,171 | 0,841 |
| KRT10 | 1418337565 | 8 | keratin, type I cytoskeletal 10 isoform X2 [Canis lupus dingo] | 0,171 | 0,841 |
| KRT10 | 928144440 | 8 | keratin, type I cytoskeletal 10 isoform X2 [Canis lupus familiaris] | 0,171 | 0,841 |
| KRT10 | 1418337567 | 8 | keratin, type I cytoskeletal 10 isoform X3 [Canis lupus dingo] | 0,171 | 0,841 |
| KRT10 | 928144442 | 8 | keratin, type I cytoskeletal 10 isoform X3 [Canis lupus familiaris] | 0,171 | 0,841 |
| LTA4H | 1418335277 | 4 | leukotriene A-4 hydrolase [Canis lupus dingo] | -0,027 | 0,841 |
| LYPD3 | 1418304786 | 5 | ly6/PLAUR domain-containing protein 3 [Canis lupus dingo] | -0,118 | 0,841 |
| LYPD3 | 73948247 | 5 | ly6/PLAUR domain-containing protein 3 [Canis lupus familiaris] | -0,118 | 0,841 |
| MARCKS | 1418348639 | 3 | myristoylated alanine-rich C-kinase substrate [Canis lupus dingo] | 0,148 | 0,841 |
| MARCKS | 928150724 | 3 | myristoylated alanine-rich C-kinase substrate [Canis lupus familiaris] | 0,148 | 0,841 |
| MSN | 545558304 | 2 | moesin isoform X1 [Canis lupus familiaris] | -0,049 | 0,841 |
| MSN | 1418243364 | 2 | moesin isoform X2 [Canis lupus dingo] | -0,049 | 0,841 |
| MSN | 928185024 | 2 | moesin isoform X3 [Canis lupus familiaris] | -0,049 | 0,841 |
| MUC5B | 1239945861 | 4 | mucin-5B isoform X1 [Canis lupus familiaris] | -0,368 | 0,841 |
| NME2 | 545510196 | 4 | nucleoside diphosphate kinase B isoform X1 [Canis lupus familiaris] | 0,170 | 0,841 |
| NQO1 | 1418308783 | 10 | NAD(P)H dehydrogenase [quinone] 1 [Canis lupus dingo] | 0,000 | 0,841 |
| NQO1 | 545500084 | 10 | NAD(P)H dehydrogenase [quinone] 1 [Canis lupus familiaris] | 0,000 | 0,841 |
| SH3BGRL3 | 345794456 | 3 | SH3 domain-binding glutamic acid-rich-like protein 3 [Canis lupus familiaris] | -0,054 | 0,841 |
| SOD1 | 1418511817 | 6 | superoxide dismutase [Cu-Zn] [Canis lupus dingo] | -0,128 | 0,841 |
| TGM3 | 1418250613 | 18 | protein-glutamine gamma-glutamyltransferase E [Canis lupus dingo] | 0,054 | 0,841 |
| HISTH4 | 1418253521 | 2 | histone H4-like [Canis lupus dingo] | 0,039 | 0,886 |
| IGH | 208342180 | 2 | immunoglobulin heavy chain variable region, partial [Canis lupus familiaris] | -0,060 | 0,905 |
| TFF3 | 1418228196 | 2 | trefoil factor 3 [Canis lupus dingo] | -0,003 | 0,905 |
| TFF3 | 156875888 | 2 | trefoil factor family peptide 3 [Canis lupus familiaris] | -0,003 | 0,905 |
| TFF3 | 157061758 | 2 | trefoil factor family peptide 3, partial [Canis lupus] | -0,003 | 0,905 |
| BPIFB1 | 1418251074 | 15 | BPI fold-containing family B member 1 [Canis lupus dingo] | 0,625 | 0,931 |
| BPIFB1 | 545540364 | 17 | BPI fold-containing family B member 1 isoform X1 [Canis lupus familiaris] | 0,444 | 0,931 |
| BPIFB1 | 57104142 | 17 | BPI fold-containing family B member 1 isoform X2 [Canis lupus familiaris] | 0,444 | 0,931 |
| FCGBP | 1418305257 | 3 | IgGFc-binding protein isoform X1 [Canis lupus dingo] | 0,040 | 0,931 |
| FCGBP | 1418305259 | 3 | IgGFc-binding protein isoform X2 [Canis lupus dingo] | -0,008 | 0,931 |
| LEG1 | 1418301038 | 7 | protein LEG1 homolog [Canis lupus dingo] | -0,090 | 0,937 |
| LEG1 | 1239885884 | 7 | protein LEG1 homolog [Canis lupus familiaris] | -0,090 | 0,937 |
| LTF | 559767226 | 2 | lactotransferrin precursor [Canis lupus familiaris] | -0,132 | 0,937 |
| PTGR1 | 1418321329 | 10 | prostaglandin reductase 1 [Canis lupus dingo] | -0,020 | 0,937 |
| PTGR1 | 545517912 | 10 | prostaglandin reductase 1 [Canis lupus familiaris] | -0,020 | 0,937 |
| OPRPN | 1418328391 | 13 | opiorphin prepropeptide [Canis lupus dingo] | -0,262 | 0,970 |
| N/A | 347943561 | 2 | cytochrome c [Canis lupus] | 0,167 | 1,000 |
| N/A | 1418270515 | 4 | poly(U)-specific endoribonuclease [Canis lupus dingo] | 0,143 | 1,000 |
| N/A | 1239968362 | 4 | poly(U)-specific endoribonuclease [Canis lupus familiaris] | 0,143 | 1,000 |
| N/A | 16607648 | 2 | unnamed protein product [Canis lupus familiaris] | 0,063 | 1,000 |
| N/A | 16607715 | 2 | unnamed protein product [Canis lupus familiaris] | 0,063 | 1,000 |
| ALB | 1418328547 | 2 | LOW QUALITY PROTEIN: serum albumin-like [Canis lupus dingo] | 0,192 | 1,000 |
| BPIFA1 | 73992235 | 4 | BPI fold-containing family A member 1 [Canis lupus familiaris] | 0,199 | 1,000 |
| CD177 | 1418304784 | 3 | CD177 antigen [Canis lupus dingo] | 0,009 | 1,000 |
| CD177 | 1239888116 | 3 | LOW QUALITY PROTEIN: CD177 antigen [Canis lupus familiaris] | 0,009 | 1,000 |
| DSG1 | 1418314253 | 11 | desmoglein-1 [Canis lupus dingo] | 0,037 | 1,000 |
| DSG1 | 545504019 | 11 | desmoglein-1 isoform X1 [Canis lupus familiaris] | 0,037 | 1,000 |
| DSG1 | 4101628 | 11 | desmoglein-1 precursor [Canis lupus familiaris] | 0,037 | 1,000 |
| ERO1A | 1418344927 | 2 | ERO1-like protein alpha [Canis lupus dingo] | 0,388 | 1,000 |
| ERO1A | 73963815 | 2 | ERO1-like protein alpha [Canis lupus familiaris] | 0,388 | 1,000 |
| FABP5 | 1418323952 | 2 | fatty acid-binding protein 5 [Canis lupus dingo] | 0,378 | 1,000 |
| GDI2 | 50978926 | 4 | rab GDP dissociation inhibitor beta [Canis lupus familiaris] | 0,086 | 1,000 |
| IGH | 208342120 | 2 | immunoglobulin heavy chain variable region, partial [Canis lupus familiaris] | 0,156 | 1,000 |
| KRT15 | 1418338550 | 5 | keratin, type I cytoskeletal 15 [Canis lupus dingo] | -0,890 | 1,000 |
| KRT24 | 1418339572 | 2 | keratin, type I cytoskeletal 24 [Canis lupus dingo] | -0,279 | 1,000 |
| KRT24 | 345805399 | 2 | keratin, type I cytoskeletal 24 [Canis lupus familiaris] | -0,279 | 1,000 |
| KRT6A | 345791910 | 7 | keratin, type II cytoskeletal 6A [Canis lupus familiaris] | -0,457 | 1,000 |
| KRT6A | 545545386 | 7 | keratin, type II cytoskeletal 6A isoform X1 [Canis lupus familiaris] | -0,457 | 1,000 |
| KRT6A | 345791839 | 7 | keratin, type II cytoskeletal 6A isoform X2 [Canis lupus familiaris] | -0,457 | 1,000 |
| KRT6A | 1418221475 | 7 | keratin, type II cytoskeletal 6A-like [Canis lupus dingo] | -0,457 | 1,000 |
| KRT6C | 1418221473 | 7 | keratin, type II cytoskeletal 6C [Canis lupus dingo] | -0,457 | 1,000 |
| KRT78 | 73996461 | 2 | keratin, type II cytoskeletal 78 [Canis lupus familiaris] | -0,110 | 1,000 |
| MUC19 | 1239968164 | 12 | mucin-19 [Canis lupus familiaris] | -0,460 | 1,000 |
| NANS | 73971430 | 2 | sialic acid synthase [Canis lupus familiaris] | 0,712 | 1,000 |
| PGAM2 | 1239883108 | 2 | phosphoglycerate mutase 2 [Canis lupus familiaris] | -0,054 | 1,000 |
| RAB1A | 55741705 | 3 | ras-related protein Rab-1A [Canis lupus familiaris] | 0,322 | 1,000 |
| RAB1B | 1418504125 | 3 | ras-related protein Rab-1B isoform X1 [Canis lupus dingo] | 0,322 | 1,000 |
| RAB1B | 1418504127 | 3 | ras-related protein Rab-1B isoform X2 [Canis lupus dingo] | 0,322 | 1,000 |
| RAB8A | 55741707 | 2 | ras-related protein Rab-8A [Canis lupus familiaris] | 0,086 | 1,000 |
| RAB8B | 1418257984 | 2 | ras-related protein Rab-8B [Canis lupus dingo] | 0,086 | 1,000 |
| SCGB1A1 | 922664320 | 4 | uteroglobin, partial [Canis lupus familiaris] | -0,056 | 1,000 |
| TFF2 | 1418228557 | 2 | trefoil factor 2 [Canis lupus dingo] | -0,189 | 1,000 |
| TFF2 | 30267901 | 4 | trefoil factor 2 [Canis lupus familiaris] | -0,189 | 1,000 |
| TFF2 | 156875886 | 4 | trefoil factor family peptide 2, partial [Canis lupus familiaris] | -0,189 | 1,000 |
